# Dynamic Allostery Modulates Catalytic Activity by Modifying the Hydrogen Bonding Network in the Catalytic Site of Human Pin1

**DOI:** 10.3390/molecules22060992

**Published:** 2017-06-15

**Authors:** Jing Wang, Ryosuke Kawasaki, Jun-ichi Uewaki, Arif U. R. Rashid, Naoya Tochio, Shin-ichi Tate

**Affiliations:** 1 Department of Mathematical and Life Sciences, School of Science, Hiroshima University, 1-3-1 Kagamiyama, Higashi-Hiroshima 739-8526, Japan; jing.wang933@gmail.com (J.W.); ryosuke-kawasaki@hiroshima-u.ac.jp (R.K.); ahmarifur@gmail.com (A.U.R.R.); 2 Research Center for the Mathematics on Chromatin Live Dynamics (RcMcD), Hiroshima University, 1-3-1 Kagamiyama, Higashi-Hiroshima 739-8526, Japan; uejun@hiroshima-u.ac.jp (J.U.); naoya-tochio@hiroshima-u.ac.jp (N.T.)

**Keywords:** dynamic allostery, human Pin1, structure dynamics, spin relaxation, hydrogen bond, H/D exchange, NMR

## Abstract

Allosteric communication among domains in modular proteins consisting of flexibly linked domains with complimentary roles remains poorly understood. To understand how complementary domains communicate, we have studied human Pin1, a representative modular protein with two domains mutually tethered by a flexible linker: a WW domain for substrate recognition and a peptidyl-prolyl isomerase (PPIase) domain. Previous studies of Pin1 showed that physical contact between the domains causes dynamic allostery by reducing conformation dynamics in the catalytic domain, which compensates for the entropy costs of substrate binding to the catalytic site and thus increases catalytic activity. In this study, the S138A mutant PPIase domain, a mutation that mimics the structural impact of the interdomain contact, was demonstrated to display dynamic allostery by rigidification of the α2-α3 loop that harbors the key catalytic residue C113. The reduced dynamics of the α2-α3 loop stabilizes the C113–H59 hydrogen bond in the hydrogen-bonding network of the catalytic site. The stabilized hydrogen bond between C113 and H59 retards initiation of isomerization, which explains the reduced isomerization rate by ~20% caused by the S138A mutation. These results provide new insight into the interdomain allosteric communication of Pin1.

## 1. Introduction

Human peptidyl-prolyl *cis*-*trans* isomerase NIMA-interacting 1 (Pin1) regulates cellular homeostasis by catalyzing *cis*-*trans* isomerization, specifically to a phosphorylated serine/threonine-proline (pS/pT-P) motif in signaling proteins [1,2,3]. Pin1 is a conformational modifier that switches the functions and the fates of pS/pT-P motif-containing proteins that play diverse cellular processes, including the cell cycle and cell growth [4]. Pin1 dysfunction is linked to an increasing number of human diseases, such as cancer and neurological disorders, including Alzheimer’s disease [5,6,7,8,9,10].

**Figure 1:**
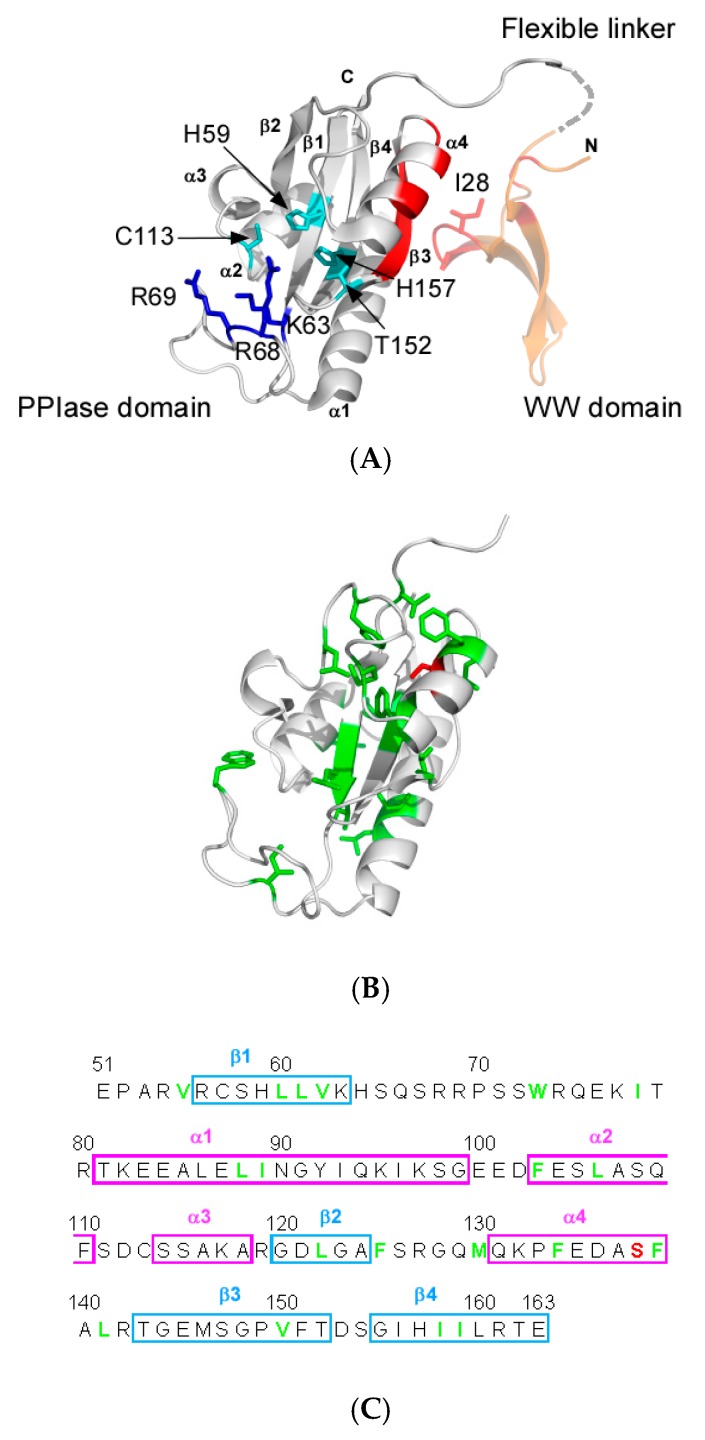
Structural features of the human Pin1 (Protein Data Bank (PDB) ID: 1PIN [11]). (**A**) Ribbon representation of the full-length Pin1 is shown. The Pin1-WW domain and the flexible linker are shown as the orange ribbon and as the dashed grey line, respectively (right-hand side). The Pin1-peptidyl-prolyl isomerase (PPIase) domain is colored grey (left-hand side). Residues involved in the central hydrogen-bonding network and in the phosphate moiety binding ‘basic triad’ are shown as cyan and blue sticks, respectively. I28 in the WW domain is also shown in stick model representation. Residues at the interdomain interface are colored red; (**B**) Solution structure of the wild-type PPIase domain [13]. The hydrophobic conduit residues and S138 are show as green and red sticks, respectively. The orientation is the same as in (**A**); (**C**) The amino acid sequence of the Pin1-PPIase domain. The positions of the α helices and β strands in the sequence are indicated by the magenta and cyan boxes, respectively. The conserved hydrophobic residues estimated by Behrsin et al. [16] are shown as green bold letters and S138 is shown as a red bold letter.

Pin1 consists of two domains: a N-terminal phosphor-peptide binding domain (WW domain) and a C-terminal catalytic domain (PPIase domain) (Figure 1A) [11]. Pin1 functions rely on interdomain communication; an isolated PPIase domain construct retains isomerase activity comparable to or greater than that of full-length Pin1, but full-length Pin1 shows different substrate binding affinity and isomerase activity [12,13,14]. In particular, cells require full-length Pin1 for viability even though the isolated PPIase domain maintains significant isomerase activity [15]. Interdomain communication between the domains in Pin1 is, therefore, functionally essential in vivo.

Previous studies demonstrated that physical contact of the WW domain to the PPIase domain changes the conformational dynamics in the catalytic site distant from the contact interface and this change in dynamics modulates enzyme activity (Figure 1A) [17]. Based on the observation, the function of Pin1 is proposed to be controlled by ‘dynamic allostery’ induced by the interdomain contact, where an allosteric effect occurs through a change in structural dynamics without conformational changes to the catalytic site [18,19].

Crystal structures of Pin1 show that the WW and the PPIase domains contact at specific sites, which include residues H27-I28-T29 (“HIT-loop”) in the WW domain and residues in α4 and β3 of the PPIase domain (Figure 1A) [11,20,21]. Nuclear magnetic resonance (NMR) experiments demonstrated that the same interdomain contact occurs transiently in solution [22]. Substrate binding to the WW domain changes the interdomain contact: some substrates strengthen the contact, whereas others diminish it to alter interdomain mobility in a substrate-dependent manner [22]. Change in the interdomain contact by substrate binding to the WW domain is functionally relevant [21]. Weakening the interdomain contact by mutation changes substrate binding affinity and isomerase activity, and also alters the conformational flexibility of the PPIase catalytic loop (residues 65–80) distal from the contact site (Figure 1A), which suggests that functional dynamic allostery arises through the interdomain contact [23].

The interdomain communication tunes the mode of dynamic allostery according to the substrate chemical structure [24,25]. Peng and coworkers showed that substrate binding to the WW domain suppresses the side chain flexibility in the sub-nanosecond time scale along a ‘conduit’ (Figure 1B) consisting of the conserved hydrophobic residues in the PPIase domain (Figure 1C) [24]. The conformational dynamics change along the conduit is shown to be stereospecific; *cis*- and *trans*-locked substrates cause different conduit responses [25]. This observation consolidates the concept for the functional linkage between the interdomain contact and the conformational dynamics in the catalytic site of the PPIase domain.

Molecular dynamics (MD) simulation demonstrated that three catalytic loops in the PPIase domain rigidify to stay in the closed form upon contact with the WW domain, which may explain the enhanced ligand affinity [26]. The simulation results advanced atomic insights into how dynamic allostery affects the catalytic site in the PPIase domain, which is composed of residues in a hydrogen bonding network, connecting residues C113–H59–H157–T152, and the basic triad (K63, R68 and R69) engaged in capturing the phosphor-moiety in the substrate [11,13,27] (Figure 1A). Intriguingly, the simulation showed that the conformational dynamics of Pin1 significantly depend on the chemical structure of the substrate bound to the WW domain as found in the above NMR experiments, but the results could not clearly describe how the changes modulate function [26]. The discussion of the entropy gain associated with reduced conformational dynamics and the selection of a conformation of the catalytic loops among the pre-organized forms seems reasonable but remains qualitative [26]. Thus, more atomic details concerning how dynamic allostery modulates the catalytic action of Pin1 are required.

Pin1 is phosphorylated at S138 by mixed-lineage kinase 3 (MLK3) (Figure 1B) [28]. The phosphorylation of S138 increases isomerase activity [28]. The phosphorylation mimicking mutant S138E showed a fourfold increase in catalytic activity relative to that of the wild-type Pin1, whereas the S138A mutant diminished this activity by about two-fold [28]. As seen from the structure of the PPIase domain, S138 locates beneath α4 in the interdomain interface (Figure 1B) [11]. The observed catalytic changes caused by altering the residue type at S138 prompted us to think that the mutations to S138 may mimic the allosteric impact to the catalytic site as that by the interdomain contact. In other words, structural characterization of the S138 mutant could aid our understanding of the dynamic allostery occurring in the catalytic domain in Pin1.

In an effort to understand the allosteric functional changes caused by S138, we initiated a structural characterization of the isolated S138A PPIase domain of Pin1. The S138A mutant showed enhanced motion for residues W73–Q75 in the catalytic loop, whilst rigidified the α2-α3 region that contacts W73 (Figure 1B). The reduced dynamics in the α2-α3 region stabilized the hydrogen-bonding between C113 and H59 over the corresponding bond in the wild-type, which was evidenced by the reduced H/D exchange rate for the imidazole ^1^H at N^ε2^ in H59. The isomerization is thought to start by disrupting the hydrogen bond between C113–H59, the stabilized hydrogen bonding at the site explains the reduced isomerization rate of the S138A mutant PPIase domain [29]. This work provides substantial details at the atomic level of the dynamic allostery that couples the interdomain interfacial site and the catalytic site, which complements previous studies exploring the roles of the interdomain contact to accomplish the elaborate interdomain communication present in Pin1 [17].

## 2. Results

### 2.1. S138A PPIase Domain is Thermally More Stable Than That of the Wild-Type

The overall structure of S138A PPIase domain determined by NMR was very similar to the wild-type structure that was also solved by NMR (Figure S1A) [13]. As indicated by chemical shift changes (Figure S1B), the S138A mutation altered the backbone structure of the β2-α4 loop (Figure S1A). This loop faces toward residue S138 in α4 (Figure S1A). Thus, the changes in the β2-α4 loop are readily ascribed to the different side chain moiety at residue 138 of the mutant. Structural statistics of the S138A PPIase domain are provided in Table S1.

**Figure 2:**
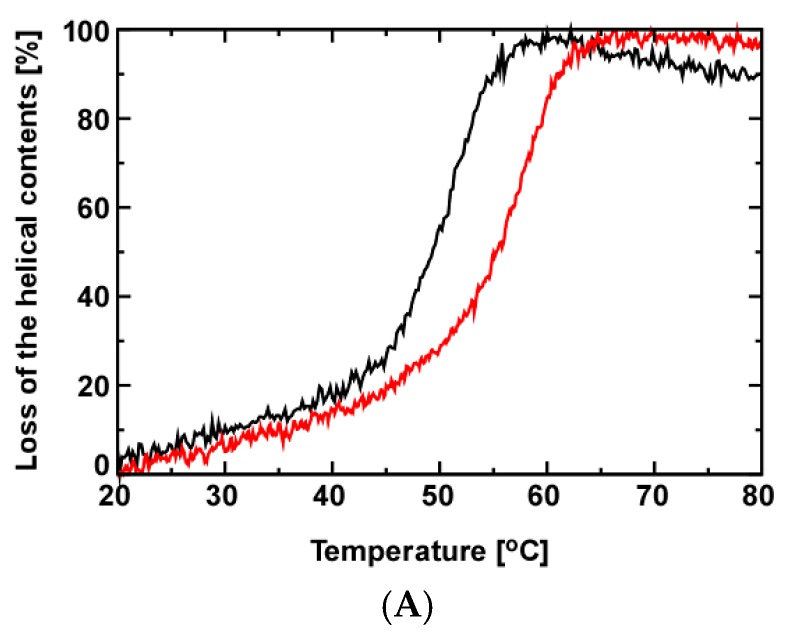

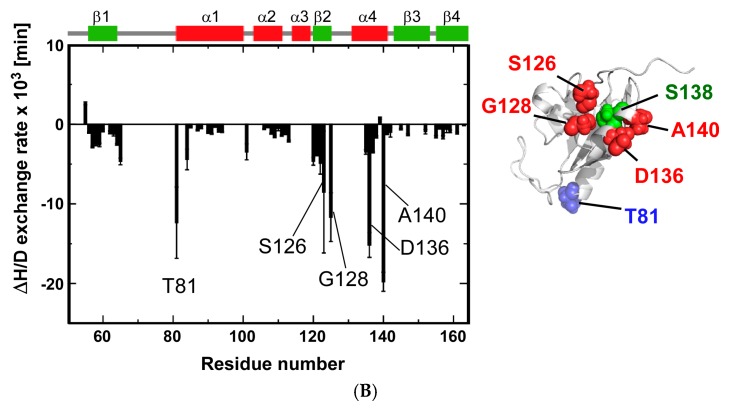
Comparison of the thermal stability and backbone amide H/D exchange rates of the wild-type [13] and S138A mutant proteins. (**A**) Thermal melting profile of the wild-type Pin1-PPIase (black) and the S138A mutant (red) by measuring the CD ellipticity at 222 nm. The definition for the loss of helical content is described in Section 4.2; (**B**) Differences in H/D exchange rates between the wild-type and the S138A mutant: the difference in H/D rate (ΔH/D rate) for each residue was obtained as ΔH/D rate = H/D rate (S138A) − H/D rate (wild-type). Only the ΔH/D rates greater than the error rages are plot. Residues without exchange rates showed no observable peaks in the first 2D ^1^H–^15^N HSQC spectrum recorded in the data series collected, implying their amide protons have rapidly exchanged to deuterons within ~10 min. Residues with reduced backbone amide H/D exchange rates in S138A are labeled. These residues near the mutation site are mapped onto the wild-type Pin1-PPIase structure as red spheres and S138 is indicated by the green sphere. The reduced amide H/D exchange rate was also found for T81 in α1, which residue is marked by blue spheres on the structure.

The thermal denaturation temperature for the S138A mutant increased by 6.4 °C relative to the wild-type (Table 1), as monitored by the change in CD molar ellipticity at 222 nm (Figure 2A). The H/D exchange experiments demonstrated that residues near the mutation site, including S126, G128, D136 and A140, had reduced H/D exchange rates when compared to the corresponding values in the wild-type, implying that residues in the mutant were more buried and thus more protected from solvent exchange (Figure 2B). The structural change of the β2-α4 loop explains the change in the H/D exchange rates (Figure S1A). Mutation of S138 to alanine loses one oxygen atom to make the residue less bulky with an increase in hydrophobicity, which should facilitate side chains of the neighboring residues of A138 to pack more tightly thereby reducing solvent exchange. The results in Figure 2B show clearly that the S138A mutation significantly rearranges the contacts among the residues near the interdomain interfacial part in the PPIase domain (Figure 1A). Besides the residues spatially neighboring to S138, T81 in α1 also significantly reduced H/D exchange rate; which residue is allosterically changed to be buried (Figure 2B). It is intriguing to note that most residues reduced their H/D exchange rates in the S138A mutant relative to the wild-type (Figure 2B): the residues may help to thermally stabilize the S138A mutant (Figure 2A).

### 2.2. The S138A Mutant Maintains Comparable Substrate Binding Ability to that of the Wild-Type but Has a Reduced Isomerization Rate

The substrate binding ability of the S138A mutant was comparable to that of the wild-type. We determined the substrate binding affinities for the wild-type and the S138A mutant with phosphor-peptide Cdc25C (EQPLpTPVTDL, where pT represents a phosphorylated threonine) using isothermal titration calorimetry (ITC) (Figure S2). In calculating the *K*_D_, the stoichiometry was fixed to 1.0 because of the limited affinities to the substrate: the site numbers (*n*) recalculated with the Δ*H* and Δ*S* values gained by the above fitting procedure were *n* = 1.000 ± 0.001 (wild) and *n* = 1.021 ± 0.036 (S138A), respectively [31,32]. *K*_D_ values for the wild-type and the S138A mutant were 1.81 ± 0.29 mM and 1.22 ± 0.88 mM, respectively (Figure S2).

**Figure 3:**
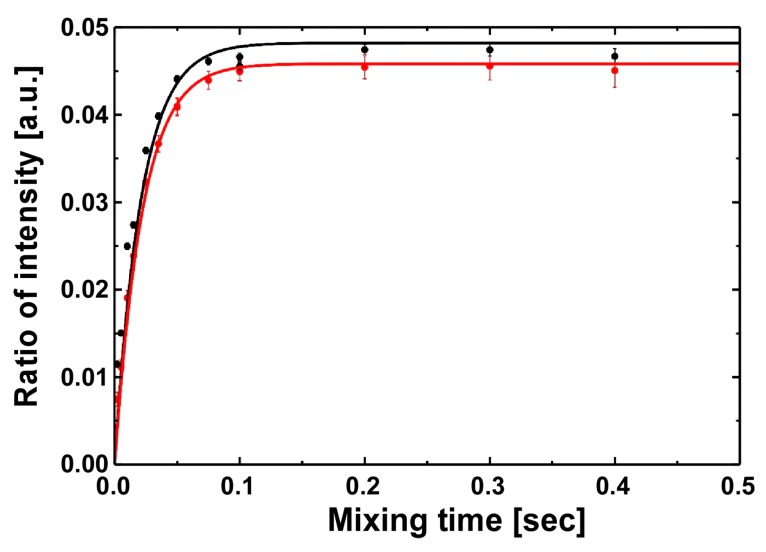
Comparison of isomerase activity between the wild-type protein and the S138A mutant to phosphorylated peptide Cdc25C. The isomerization rate for the S138A mutant was determined by a series of EXSY spectra with the phosphor-peptide Cdc25C at varying exchange times (red). The previously reported data for the wild-type [13] were included for reference (black).

The isomerization rates were directly determined by NMR with Exchange Spectroscopy (EXSY) experiments for the S138A mutant using Cdc25C as the substrate (Figure 3) [13,23,33]. The isomerization rates for the S138A mutant are compared with the values for the wild-type and other mutants in Table 1 [13,30]. Despite the less significant difference in the substrate binding affinities between the proteins, the S138A mutant had reduced isomerization rates by ~20% in the net exchange rate *k*_EX_ (*k*_EX_ = *k*_CT_ + *k*_TC_).

The S138A mutant retains the same catalytic site structure as the wild-type protein (Figure S1A), which is also supported by the marginal chemical shift changes to key residues in the basic triad (K63, R68 and R69) and the hydrogen-bonding network (C113–H59–H157–T152) (Figure S1B). This observation differs from the result on the C113D mutant PPIase domain. Here, the C113D mutant altered the catalytic β1-α1 loop structure to have shown the reduced affinity towards the substrate and the diminished isomerization rates (Table 1) [13]. Since no clear structural changes to the catalytic site of the S138A mutant were observed and thus the mutant retained the comparable substrate binding ability to the wild-type, as evident in the ITC experiments, the moderate reduction in its isomerization activity should be ascribed to the change in the structure dynamics in the catalytic site. The S138A mutant is, therefore, realized to mimic the interdomain contact to cause the dynamic allostery observed in the full-length Pin1 [17].

### 2.3. Conformational Dynamics of the S138A Mutant Revealed by ^15^N Nuclear Spin Relaxation Analysis

We compared structural dynamics between the wild-type and S138A PPIase domains by the reduced spectral density approach [34]. The uniform distribution of ^15^N–^1^H hetero-nuclear Overhauser effect values (hNOEs) above 0.8 for residues other than those at the N-terminus and α1-α2 loop (Figure S3A) shows that most residues in the wild-type and S138A PPIase domains have limited internal motion on the picosecond time scale. Backbone amide bond motions in most residues of the PPIase domain are, therefore, dominated by a single motion defined by the overall rotational correlation time, *τ_c_* [35]. Under this condition, there holds the correlation between *J*(0) and the spectral density at *ω_N_* as below [34]:

(1) $$J\left(\omega_{N}\right)=\frac{J\left(0\right)}{1+6.25{\left(\omega_{N}J\left(0\right)\right)}^{2}}$$

The graphical representation of the relation in Equation (1) is shown in Figure S4. Data points for residues with N–H bond motions singly dominated by *τ_c_* are near or on the solid line (Figure S4, point *a*). Residues with significant rapid internal motion are defined by the local correlation time, *τ_e_*, and give rise to data points well below the solid line (Figure S4, point b): *τ_e_* and *τ_c_* are in the ps and ns timescale regimes, respectively [34]. In the extreme case for residues with the N–H bond motion mainly dominated by rapid internal motion yield data points on the opposite slope to point *a* (Figure S4, point *c*; where 1/*τ_l_* = 1/*τ_c_* + 1/*τ_e_*). Residues with slow conformational exchange on the µs–ms timescale have larger *J*(0) values and give rise to outlier points well above the solid line (Figure S4, position *d*) [34].

#### 2.3.1. The S138A Mutant Becomes Compact in Shape Relative to the Wild-Type PPIase Domain

**Figure 4:**
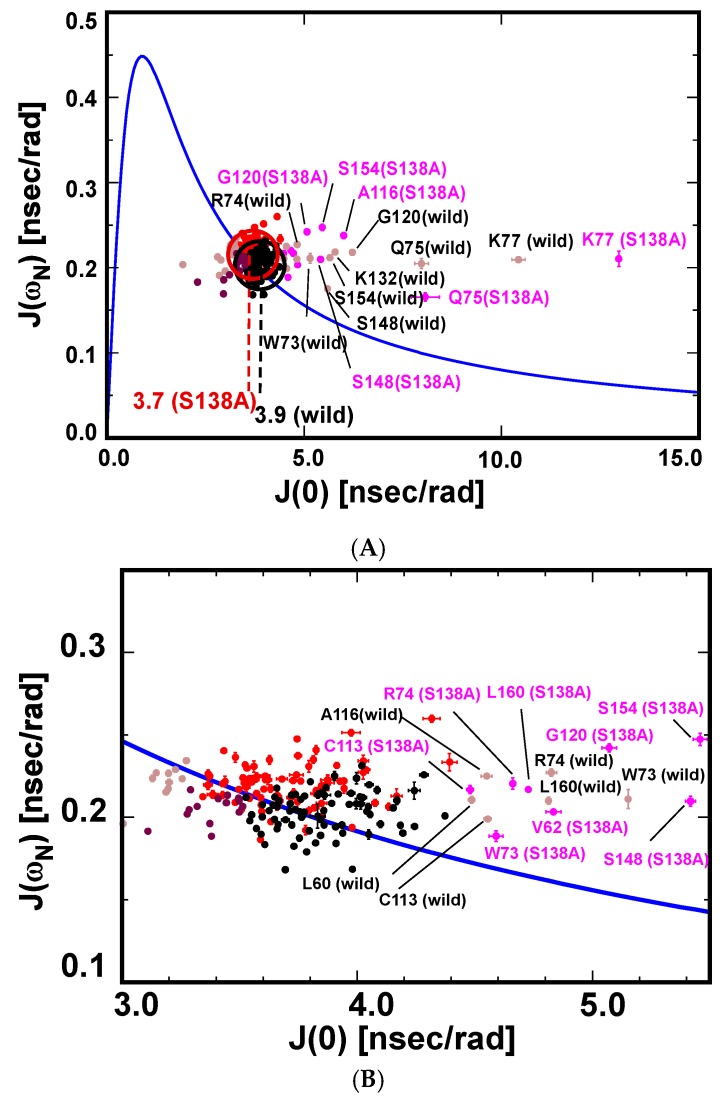
Graphical correlations between *J*(0) and *J*(*ω_N_*) for the wild-type Pin1-PPIase and S138A mutant. (**A**) The simple correlation represented by Equation (1) is shown by the blue curve. The experimentally obtained *J*(0)–*J*(*ω_N_*) correlations for residues in the wild-type Pin1-PPIase and S138A mutant are plotted. Residues with *J*(0) values within the average *J*(0) ± 0.5σ for the wild-type and S138A mutant are colored black and red, respectively. Residues with *J*(0) values outside the above range for the wild-type and S138A mutant are colored light brown and purple, respectively; (**B**) An expanded view of the clustered data points in (**A**).

The *J*(0)–*J*(*ω_N_*) correlations for residues in the wild-type protein and the S138A mutant are plotted on the solid line representing Equation (1) (Figure 4A). As envisaged from the hNOE values (Figure S3A), most residues in the wild-type and the S138A mutant are clustered on the solid line: the clustered residues have N–H motions solely defined by *τ_c_* (Figure 4A). The average *J*(0) values for residues that are dominated by *τ_c_* in the wild-type and the S138A mutant were 3.9 ± 0.2 ns/rad and 3.6 ± 0.2 ns/rad, respectively (Figure 4A). The number of residues with *J*(0) values within the average *J*(0) ± 0.5σ (σ, standard deviation for all the observed *J*(0) values) was 79 of a possible 108 residues in the wild-type and 79 of a possible 104 residues in the S138A mutant. The re-averaged *J*(0) values for the selected residues were 3.9 ± 0.2 ns/rad (wild-type) and 3.7 ± 0.2 ns/rad (S138A mutant), respectively (Figure 4A). *J*(0) values give the overall rotational correlation times, *τ_c_*, as 9.8 ± 0.5 ns (wild-type) and 9.3 ± 0.5 ns (S138A mutant). The lower *τ_c_* value for the S138A mutant shows that this mutant is smaller in molecular volume when compared with that of the wild-type PPIase domain (Figure 4A).

#### 2.3.2. Changes in Conformational Exchange on the µs–ms Timescale by the S138A Mutation

Some residues in the PPIase domain show significant conformational exchange on the µs–ms timescale, as determined by their corresponding correlations lying well away from the theoretical curve for residues with N–H motion dominated by a single correlation time (Figure 4). Inspection of the data reveals that the wild-type and the S138A mutant have 12 and 11 residues, respectively, displaying such motional properties; these residues were selected because they have *J*(0) values over the average *J*(0) + 0.5σ (Figure 4).

**Figure 5:**
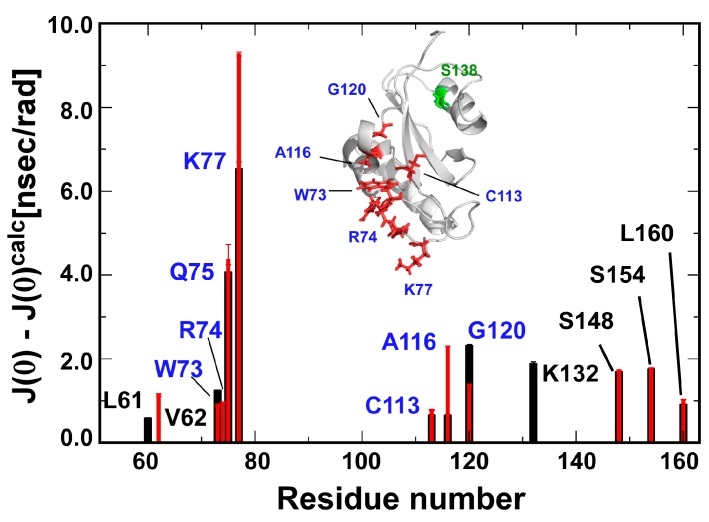
Residue-wise differences between the experimentally obtained *J*(0) and the calculated *J*(0) (*J*(0)^calc^) are shown; only the residues outlying over the theoretical curve are considered (Figure 4). The definition for *J*(0)^calc^ is described in the main text. The differences for the wild-type and S138A mutant are shown in black and red, respectively. Residues with allosteric changes induced by S138A mutation are labeled. The structural positions for these residues and S138 are modeled in the wild-type Pin1-PPIase structure [13] as red and green sticks, respectively. Residues focused in the text are marked in blue, which residues are also marked on the solution structure of the S138A mutant PPIase domain (inset).

The conformational exchange contributing to *J*(0) was estimated by a difference between the experimental *J*(0) and the calculated *J*^calc^(0) using the *τ_c_* (Figure S4). Residues that have significant magnitude of internal motion defined by *τ_e_* generally have *J*(0) values that can be described by two correlation times as:

(2) $$J\left(0\right)=\frac{2}{5}S_{0}\tau_{c}+\left(1-S_{0}\right)\frac{2}{5}\tau_{l}$$

where *S*_0_ (0 < *S*_0_ < 1.0) is used for scaling to the contribution from rapid internal motion, which is equivalent to the square order parameter, *S*^2^, in the Lipari-Szabo formalism [36]. The correlation time *τ_l_* is defined by two correlation times as 1/*τ_l_* = 1/*τ_c_* + 1/*τ_e_* [36]. As envisaged from Equation (2), the value *J*(0)–*J*^calc^(0) estimated by assuming a single tumbling motion would underestimate the conformational exchange contribution to *J*(0), if the residue had a significant magnitude of internal motion (Figure S4). In the case of the PPIase domains of the wild-type and the mutant, however, the bias should be negligible for most residues, because residues with hNOEs >0.80 should have *S*_0_ values >0.9, giving an error that can reach a maximum of 10% when compared with the corresponding residues between two proteins. We, therefore, directly compared the values *J*(0)–*J*^calc^(0) between the wild-type and S138A mutant without further correction to the values (Figure 5); in which only the residues significantly outlying over the theoretical curve were focused (Figure 4).

The residues W73, R74, Q75 and K77 in the catalytic β1-α1 loop display prominent conformational exchange on the µs–ms timescale when compared with the other residues of both the wild-type and the S138A mutant (Figure 5). The change in the conformational exchange for the residues in β1-α1 loop was marginal in the S138A mutant. In contrast to those residues, A116 in α3 showed enhanced motion, while G120 in β2 showed a reduction in motion on the µs–ms timescale (Figure 5) The presence of NOE connectivities between the side chain of W73 and residues in α2 and α3 clearly indicates that these residues form contact with each other and are in close proximity to each other (Figure S1A inset and Figure S1C). The change in dynamics to residue A116 and presumably G120 might be evoked via W73 because of the change in motion of the segment including residues 73–75 in the catalytic β1-α1 loop, which residues are intrinsically dynamic in motion (Figure 5).

#### 2.3.3. S138A Mutation Changes the Backbone Dynamics of the Catalytic Site Residues

**Figure 6:**
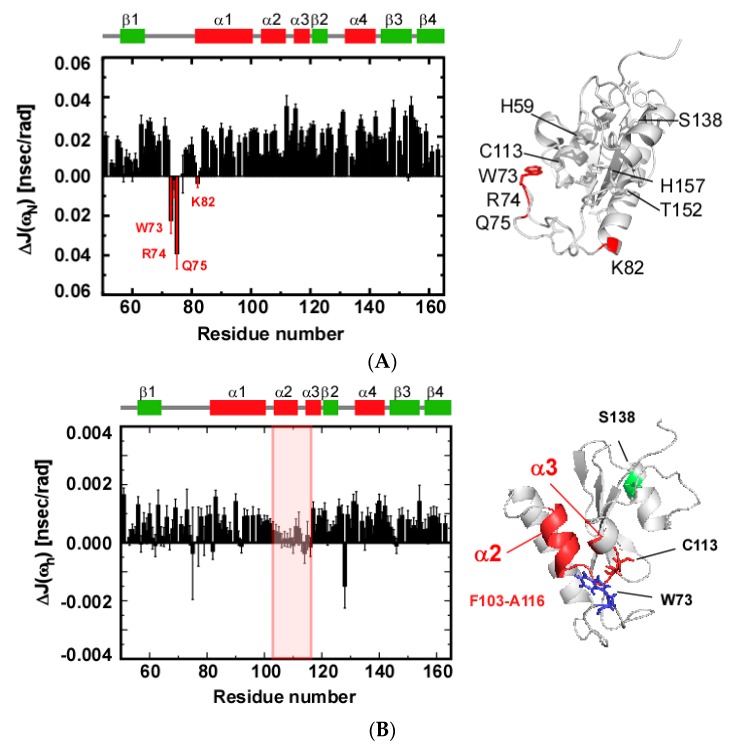
S138A mutation induced changes in structural dynamics. (**A**) Differences in spectral density function *J*(*ω_N_*). The difference was calculated for each residue as Δ*J*(*ω_N_*) = *J*(*ω_N_*)^S138A^ − *J*(*ω_N_*)^wild−type^, where *J*(*ω_N_*)^S138A^ and *J*(*ω_N_*)^wild−type^ are the values for the S138A mutant and the wild-type protein [13], respectively. Residues with negative values are labeled and marked in red. In the left panel, residues with negative Δ*J*(*ω_N_*) are colored red. The conserved hydrophobic residues, S138 and the hydrogen-bonding network residues are modeled as sticks. (**B**) Differences in spectral density function *J*(*ω_h_*). The difference was calculated for each residue as Δ*J*(*ω_h_*) = *J*(*ω_h_*)^S138A^ − *J*(*ω_h_*)^wild−type^, where *J*(*ω_h_*)^S138A^ and *J*(*ω_h_*)^wild−type^ are the values for the S138A mutant and the wild-type protein [13], respectively. The region, F103–A116, with values lower than other parts is orange-boxed (right). The region, F103–A116, W73 and S138 are shown in red, blue and green, respectively (left).

The differences between wild-type and the S138A spectral densities *J*(*ω_N_*) and *J*(*ω_h_*) uncovered other aspects of changes in conformational dynamics induced by the S138A mutation (Figure 6).

The differences in *J*(*ω_N_*) calculated as *J*(*ω_N_*)^S138A^ − *J*(*ω_N_*)^wild−type^ for most residues in S138A mutant were positive due to the smaller *τ_c_* value of the mutant (Figure 6A); residues in a protein tumbling with a smaller *τ_c_* tend to have greater *J*(*ω_N_*) values, if the residue has minimal internal motion (Figure 4A). W73, R74, Q75 and K82, are outliers because these residues gave rise to negative Δ*J*(*ω_N_*) values (Figure 6A), which indicates that these four residues of the catalytic β1-α1 loop display larger internal motions for their N–H bonds on the ps timescale in the S138A mutant (Figure 6A).

The Δ*J*(*ω_h_*) values defined as *J*(*ω_h_*)^S138A^ − *J*(*ω_h_*)^wild−type^ showed a profile that did not correlate with that of Δ*J*(*ω_N_*) (Figure 6B). Residues F103–A116 located in α2 and α2-α3 loop characteristically had reduced values when compared with other residues. It is noted that the positively biased distribution of Δ*J*(*ω_h_*) values over the residues is also ascribed to the smaller *τ_c_* for the S138A mutant [34].

The difference in hNOEs between the wild-type and S183A mutant (hNOE^S138A^ − hNOE^wild−type^) showed that the residues F103–A116 are outliers that have increased hNOE values (Figure S3B). The reduced *τ_c_* for the S138A mutant makes ΔhNOEs systematically negative for residues.

The correlated profiles between the Δ*J*(*ω_N_*) and ΔhNOE consistently suggest that the region comprising residues F103–A116 reduced their internal dynamics on the timescale ranging from sub-ns to a few ns in the S138A mutant [37].

#### 2.3.4. S138A Mutation Caused Dynamic Allostery in the PPIase Domain

The ^15^N nuclear spin relaxation analyses described above demonstrated that the S138A mutation caused significant changes in conformational dynamics to residues in the catalytic site (Figure 6). The residues in the catalytic β1-α1 loop showed enhanced motion on the ps timescale (Figure 6A), while residues in the α2 and α2-α3 loop had diminished motion on the sub-ns to low ns time regime (Figure 6B). These two regions are in physical contact mediated by the indole ring of W73 (Figure S1A,C). Comparison of the structures between the wild-type and the S138A mutant PPIase domains showed no apparent structural changes observed in the catalytic site consisting of the above regions of the domain, although the conformation of the β2-α4 loop near the mutation site did change (Figure S1A).

It should be remarked here that the S138A mutation near the interdomain contact site of Pin1 caused dynamic allostery in the sense by Cooper and Dryden; in which conformational dynamics change at the catalytic site with keeping its conformation [18,19]. Which prompted us to proceed to see how the S138A mutation modulates the isomerization activity through the dynamic allostery to the catalytic site.

### 2.4. Change in the Hydrogen Bonding Network in the Catalytic Site Caused by the S138A Mutation

The hydrogen bonding network of the wild type PPIase domain is positioned across the catalytic site, which connects residues C113–H59–H157–T152 (Figure 1A) [27]. We previously assessed the strength of the hydrogen bonds in the network by measuring the H/D isotope effect to the chemical shifts of ^15^N nuclei in the imidazole rings of H59 and H157 [13,30].

**Figure 7:**
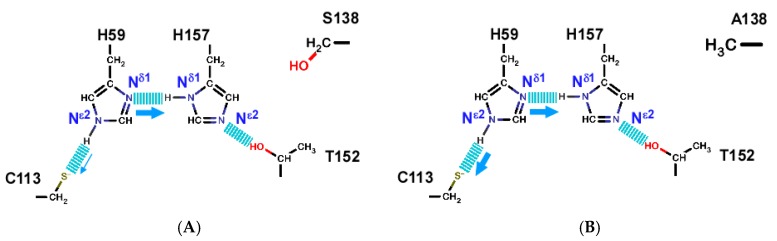
Allosteric changes to the hydrogen-bond network in Pin1-PPIase induced by the S138A mutation. (**A**) The hydrogen-bond network identified in the wild-type Pin1 PPIase according to the high-resolution crystal structure of Par14 [27]. H59 forms a stronger hydrogen bond to H157 than to C113, as previously reported [13]; (**B**) The S138A mutation strengthens the hydrogen bond between H59 and C113 while maintaining a strong hydrogen bond to H157. The thickness of the arrow represents the strength of hydrogen bond; thicker arrow means stronger bond.

The tautomeric states of the H59 and H157 in the S138A mutant were determined by multi-bond ^1^H–^15^N hetero-nuclear single quantum coherence (HSQC) spectra (Figure S5) [38], with the results showing that H59 N^ε2^ and H157 N^δ1^ are protonated. The orientations of the imidazole rings for H59 and H157 were determined by NOEs, showing H59 and H157 maintain the same orientations as in the wild-type [13]. The S138A mutant, therefore, retains the same hydrogen-bonding network as in the wild-type (Figure 7A).

**Figure 8:**
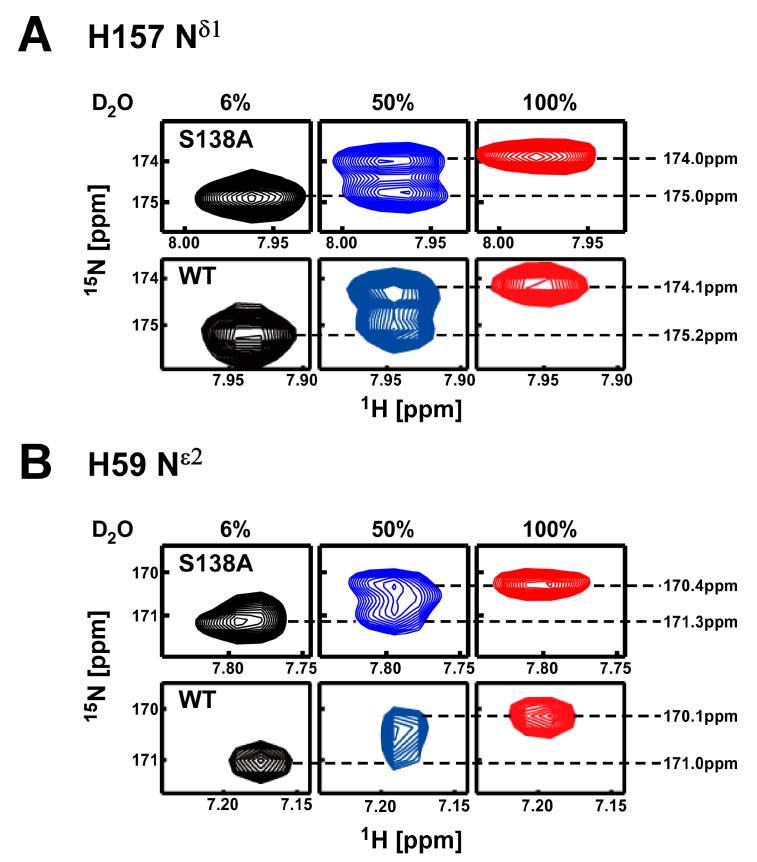
H/D isotope effects on the chemical shifts for ^15^N nuclei in histidine imidazole rings for the S138A Pin1-PPIase mutant were measured in solutions with different D_2_O contents. The spectra for the S138A mutant are compared with those of the wild-type reported previously [13]. (**A**) 2D ^1^H–^15^N^δ1^ hetero-nuclear single quantum coherence (HSQC) signals for H157 collected in solutions containing 6% (black, left), 50% (blue, middle) and 100% D_2_O (red, right); (**B**) Same data set for 2D ^1^H–^15^N^ε2^ signals of H59. In each data, upper panel is for the S138A mutant and the lower is for the wild-type shown for comparison [13].

H59 N^ε2^ and H157 N^δ1^ in the 138A mutant showed clear deuterium isotope shifts in a 100% D_2_O solution (Figure 8). As we reported previously, the imidazole ^15^N signal shape observed in 50% D_2_O solution changes according to the hydrogen exchange rate of the bound proton at the imidazole ^15^N atom. Thus, the signal shape monitors the strength of the hydrogen bond formed by the imidazole ring [13,30]. If the hydrogen exchange at ^15^N imidazole atom is sufficiently slow (strong hydrogen bond), two discrete signals from ^15^NH and ^15^ND components are observed, thus giving a doublet (Figure 8A). Conversely, if hydrogen exchange is sufficiently fast (weak hydrogen bond), the ^15^N gives a singlet resonating at the population weighted average position of the ^15^NH and ^15^ND chemical shifts (Figure 8B).

The imidazole ^15^N signals for both H59 N^ε2^ and H157 N^δ1^ in the S138A mutant were doublets in 50% D_2_O solution (Figure 8). The hydrogen bonds formed by H157 N^δ1^ and H59 N^ε2^ in the S138A mutant are stable enough to suppress ^1^H exchange (Figure 7B). In contrast to the result for the S138A mutant, the wild-type imidazole signals in 50% D_2_O solution for H157 N^δ1^ and H59 N^ε2^ showed doublet and singlet signals, respectively (Figure 8). The hydrogen bond between H59 and H157 is stable (slow hydrogen exchange), whereas the hydrogen bond between H59 and C113 is labile (fast hydrogen exchange) in the wild-type protein (Figure 7A) [13].

The backbone structure of the S138A mutant showed no apparent structural changes in the catalytic site (Figure S1). However, the hydrogen-bonding network was stabilized allosterically by the S138A mutation, with maintaining the side chain arrangement of H59 and H157 in the network (Figure 7B). The stabilized hydrogen-bonding network contributes to the increased thermal stability observed for the S138A mutant (Figure 2A).

## 3. Discussion

### 3.1. Envisaged Functional Regulation through Dynamic Allostery in Pin1

Multidomain proteins like Pin1 are abundant in nature [39], in which consecutive globular domains are linked by unstructured flexible linkers, also referred to as intrinsically disordered regions (IDRs) [40]. In such modular proteins, the linked domains should have well-coordinated actions through some interdomain communication, but how they cooperate with each other remains essentially unresolved.

Pin1 is a well-characterized modular protein, in which interdomain communication occurs through physical contact between the WW and PPIase domains (Figure 1A). Substrate binding to the WW domain changes the physical contact of the domains and leads to rigidification of side chains of residues along a ‘conduit’ linking the interdomain interface to the catalytic site (Figure 1B). Previous MD simulations demonstrated that the interdomain contact supplemented by the substrate bridging the interdomain interaction reduced the dynamics of the three catalytic loops (residues 63–73, 126–132 and 151–155) such that these regions adopted a compact form in the PPIase domain [26].

The above studies consistently indicated that the interdomain contact reduces conformational dynamics in the catalytic site of the PPIase domain [17]. The reduced structural dynamics in the active site provides an entropic gain in binding to the substrate with selecting a conformation that is presumably advantageous for capturing substrate. Such a mechanism may explain the enhancement of substrate binding by the PPIase domain in the presence of the WW domain [26]. The proposed mechanism is in agreement with dynamic allostery [19], in which the change in structural dynamics at a distal site plays a functional regulatory role without altering the structure.

Data on Pin1 provide a consensus view into the mechanism of catalytic activation, in which interdomain communication of Pin1 via physical contact between the two domains regulates isomerase activity through the dynamic allosteric coupling between the contact interface and the catalytic site [17]. MD simulations support the dynamic allostery regulates catalytic activity in Pin1, but these simulations do not clearly show how the dynamic allostery controls the catalytic action at atomic level [26,41].

### 3.2. S138A Mimics the Interdomain Contact and Enables Exploration of the Role of Dynamic Allostery

The challenge in experimentally exploring the role of the dynamic allostery in atomic detail is due to the observation that the interdomain contact is transient. The inability in keeping the domains in contact has limited NMR analysis of chemical shift perturbations and changes in dynamic parameters [17,24,25]. In this work, we used the S138A mutant to mimic the impact of the interdomain contact.

The idea in choosing the point mutant arises from a report that the phosphorylation of S138 enhances isomerase activity of Pin1 [28]. S138 locates in α4 that constitutes the interdomain interface (Figure 1B). The phosphor-serine mimic mutation S138E was confirmed to show four-fold enhanced activity in vivo and in vitro relative to the wild-type Pin1 [28]. This report prompted us to hypothesize that the S138E PPIase domain would mimic the catalytic domain activated by the interdomain contact.

The isolated S138E PPIase domain was used because we could avoid the requirement of the WW domain interaction with the PPIase domain. Attempts to obtain the S138E PPIase domain for the NMR analysis failed. We applied various approaches for obtaining the sample by changing the vectors, expression and purification protocols, but none of them was successful. For unknown reasons the expression of the S138E PPIase domain in *Escherichia coli* was extremely low. As a second sample choicef, we tried to get full-length Pin1 with the S138E mutation. The full-length Pin1 with the S138E mutation was purified. However, NMR spectra of the sample showed that the full-length Pin1 mutant was unfolded at 26 °C and even lower temperatures (data not shown). We think the S138E mutation makes the PPIase domain too unstable and this instability impedes NMR analysis of the sample.

As an alternative to S138E, we decided to work on the S138A mutant that is known to lose about 50% activity relative to the wild-type [28]. We could successfully produce and isolate the S138A mutant PPIase domain, as in the cases for other mutant PPIase domains [13,30]. Because of the reported reduction in the activity for the S138A mutant Pin1 in vivo and in vitro [28], the S138A was expected to allosterically transmit the impact at the S138 to the catalytic site: S138 is near the contact interface in the PPIase domain (Figure 1B).

The predicted dynamic allostery in the S138A mutant PPIase domain was found to be valid in the present work. Here, the S138A mutation caused significant changes to the conformational dynamics of residues within the catalytic site with limited impact on the structure. Although our dynamics changes yielded a negative effect to the catalytic rate, the S138A mutant PPIase mimics the interdomain contact state to cause dynamic allostery to modulate catalytic activity. The present analysis, therefore, gives a model to understand how dynamic allostery changes the catalytic action at the atomic level.

### 3.3. Dynamic Allostery Experimentally Observed in the S138A Mutant

The structure of the S138A mutant PPIase showed changes to the β2-α4 loop when compared with the wild-type structure. This was also indicated by changes in the chemical shifts when compared with those of the wild-type protein (Figure S1). The structural change in the loop appears to be caused by the rearrangement of residue-residue contacts near the mutation site S138. The reduced H/D exchange rates for the amide protons of S126, G128, D136 and A140, all of which are spatially neighboring to S138, provide evidence that significant changes in residue-residue contacts made the four residues being more protect from solvent when compared with the wild-type protein (Figure 2B).

The other loops in the catalytic site including β1-α1 and α2-α3 did not show apparent structural changes in the S138A mutant, which is supported by the marginal chemical shift changes for residues in these regions of the protein (Figure S1). However, these two loops showed significant changes to their conformational dynamics (Figure 6). It is intriguing to note the change in their dynamics occurred on different timescales: residues W73, R74, Q75 and K82 in the β1-α1 loop showed enhanced internal motions on the ps timescale, whereas residues in α3 and α2-α3 loop displayed reduced motion on the sub-ns and low ns timescale (Figure 6). These two regions physically contact through the side chain of W73 (Figure S1). These two parts also have significant conformational dynamics on the μs–ms timescale, and residues in these parts have changes in their dynamics in the S138A mutant (Figure 5).

The above observation describing changes in conformational dynamics of the loops β1-α1 and α2-α3 over a wide range of timescales caused by the S138A mutation are hypothesized to represent dynamic allostery, because the loops showed no apparent structural changes in the mutant when compared with the structure of the wild-type protein (Figure S1). We, therefore, claim the present experiments on the S138A mutant PPIase domain provide atomic insights into the dynamic allostery that has been suggested from previous studies [17].

Microsecond-long molecular dynamics (MD) simulations have revealed the change in the dynamic contacts among residues in the PPIase domain upon binding to the substrate [41]. The simulations identified residues that gained more dynamic contacts and lost contacts during the microsecond-long dynamics in the presence of the substrate [41]. The results showed that residues with changes in dynamic residue-residue contact are clustered near S138 and the β1-α1 and α2-α3 loops; in the simulation, some intervening residue-residue contacts bridge the two distinctively clustered parts to make them allosterically coupled [41].

**Figure 9:**
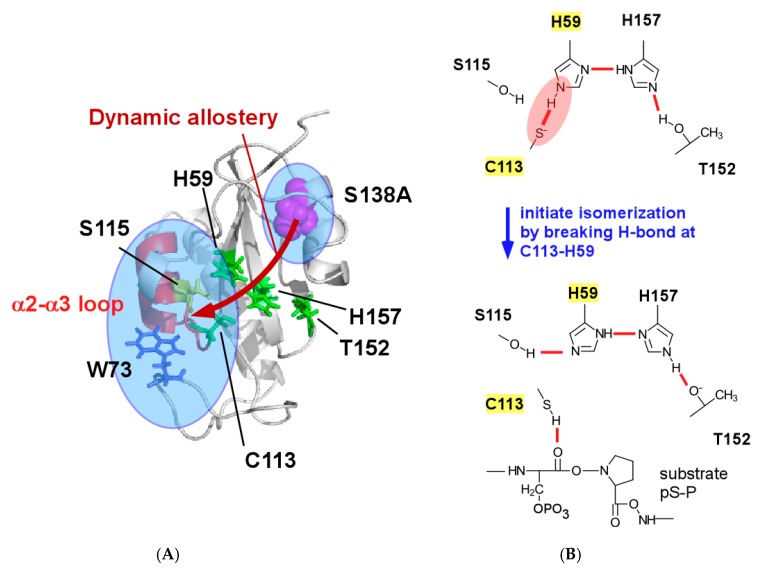
Dynamic allostery caused by the S138A mutation in the PPIase domain retards the isomerization process. (**A**) The S138A mutation rearranges residue-residue contacts among residues at the interdomain interface, marked with small blue circles. The change in residue contacts near the mutation site cause the dynamic allostery to rigidify the α2-α3 loop colored in red; (**B**) The reduced dynamics for the α2-α3 loop strengthens the hydrogen bond formed by the S^γ^ thiolate of C113 to N^ε2^ in the imidazole ring of H59 (top). The isomerization is supposed to rearrange the hydrogen bonds among residues in the hydrogen-bonding network upon binding to substrate, which may be initiated by the breaking of the hydrogen bond between C113 and H59 due to protonation of the thiolate in C113 (bottom) [29]. The strengthened hydrogen bond between C113 and H59 in the mutant when compared with that in the wild-type retards this initiation process because of the inefficient rupture of the C113–H59 hydrogen bond.

As evident by the changes in the H/D exchange rates for residues near S138 (Figure 2B) with the structural change of the β2-α4 loop (Figure S1), residue–residue contacts among those residues should have been significantly disturbed by the mutation. The change in residue–residue contacts by the S138A mutation is not exactly the same as that posed by the interdomain contact. The mutation, however, does rearrange residue-residue contacts at the interdomain interface to excite dynamic allostery at the distal active site loops, β1-α1 and α2-α3, in a closely resembling manner to the changes in the residue-residue contacts by the interdomain contact found in the MD simulations (Figure 9A) [41]. The coincident observation between experimental and theoretical results further strengthens the significance of dynamic allostery observed in the S138A mutant; which occurs in essentially the same mechanism as for the allosteric effect initiated by the interdomain contact in full-length Pin1.

### 3.4. Dynamic Allostery Changes the Hydrogen-Bonding Network

The dynamic allostery in the S138A mutant strengthened the hydrogen bond between C113 and H59 (Figure 7). In the wild-type PPIase domain, the corresponding hydrogen bond is weaker than that in the S138A mutant, while the hydrogen bond between H59–H157 is as stable as the corresponding bond in the mutant, which was shown by the shapes of the imidazole ^15^N signals in 50% D_2_O solution for H59 and H157 [13]. The strengthened hydrogen bonding in the network in the S138A may contribute to increase the thermal stability of the S138A mutant (Figure 2A).

As discussed above, C113 is located in the α2-α3 loop and the loop is in contact with the side chain of W73 (Figure S1A). The α2-α3 loop in the S138A mutant became rigidified by reducing motion on the sub-ns and low ns timescales, as revealed by the changes in hNOEs (Figure S3B) and *J*(*ω_h_*) values (Figure 6B). The reduced conformation dynamics of the α2-α3 loop could restrict C113 to ensure formation of the hydrogen bond to H59 over a narrow distance range; the conformational fluctuation of the α2-α3 loop varies the distance between C113 and H59 to alleviate hydrogen bonding between these two residues in the wild-type.

The strengthened hydrogen bond between C113 and H59 stabilized the structure of the PPIase domain (Figure 2A), but it reduced the isomerization reaction. Barman and coworkers have suggested that the coordinated change in protonation among residues in the hydrogen bonding network should occur in the isomerization of the pS/pT-P motif (Figure 9B) [29]. They theoretically proposed that C113 should remain in an unprotonated state in the apo-form of the PPIase domain and the thiolate is stabilized by hydrogen bonding with H59 (Figure 7) [29]. Upon binding the substrate to the catalytic site, C113 may become protonated to rearrange a hydrogen bond to the carbonyl oxygen of the phosphor serine or threonine residue in the substrate, with simultaneous formation of a hydrogen bond between S115 to H59, which also changes the tautomeric state of H59 to become protonated at N^δ1^ from N^ε2^ [29]. In their proposed mechanism, the protonation to the thiolate of C113 with breaking the hydrogen bond between C113 and H59 due to binding of the substrate is the pivotal step to initiate isomerization [29]. According to the mechanism, the stabilization of the hydrogen bond between C113 and H59 should block the pivotal step in protonating the thiolate of C113, which could explain the reduced isomerization activity of the S138A mutation by ~20% (Table 1).

## 4. Materials and Methods

### 4.1. Pin1 Sample Preparation

The cDNA encoding human Pin1 PPIase domain was cloned into the pET28a expression vector (Addgene Inc., Cambridge, MA, USA), as reported previously [13,30]. Site-directed mutagenesis to yield the S138A mutation in the Pin1 PPIase domain was performed by KOD FX (Toyobo, Osaka, Japan). The expression and purification of the S138A Pin1 PPIase mutant was carried out as described previously [13,30].

### 4.2. Measuring the Thermal Stability of the S138A PPIase Domain

The change in the molar ellipticity at 222 nm (*θ*_222,obs_) was used to monitor the thermal denaturing process of the proteins using a 720 W spectrometer (JASCO, Tokyo, Japan) and a 1 mm quartz cell. The sample buffer consisted of 50 mM sodium phosphate (pH 6.0), 100 mM Na_2_SO_4_ and 1 mM dithiothreitol (DTT). The protein concentration was adjusted to 2 µM. The temperature range used was 20–80 °C, with an increment rate at 1 °C/min. The loss of helical content was calculated as follows:

(3) $$\mathrm{Loss}\;\mathrm{of}\;\mathrm{helical}\;\mathrm{contents}\left[\%\right]=\frac{\theta_{222,\mathrm{obs}}-\theta_{222,\min}}{\theta_{222,\max}-\theta_{222,\min}}\times 100$$

where *θ*_222,max_ and *θ*_222,min_ are the maximum and minimum *θ*_222_ values in the range of 20−80 °C, respectively.

### 4.3. NMR Spectroscopy and Structure Determination

The structure of the S138A Pin1 PPIase domain was determined according to standard NMR methods using a uniformly ^13^C/^15^N-labeled sample [42]. All NMR data were collected on an Avance II spectrometer (Bruker, Billerica, MA, USA) equipped with a triple-resonance cryogenic probe operating at a ^1^H resonance frequency of 700 MHz. The protein sample was dissolved in a buffer solution consisting of 50 mM sodium phosphate (pH 6.6), 100 mM Na_2_SO_4_, 5 mM EDTA, 1 mM DTT and 0.03% NaN_3_. The protein concentrations were 1.0 mM and the sample temperature was 299 K for all NMR experiments, unless otherwise noted. Data processing and analysis were performed with the programs NMRPipe [43] and KUJIRA [44] running with NMRview [45], respectively. The CYANA utility was used to obtain automatic NOE assignments used in the structure determination step [46,47]. Backbone dihedral angle restraints were generated with TALOS+ [48]. The 50 lowest-target function CYANA structures were subjected to explicit water refinement using the program XPLOR-NIH 2.31 with distance and dihedral restraints [49]. The 10 lowest-energy structures resulting from the calculation with XPLOR-NIH 2.31 were validated according to the recommended procedure [50]. The structural statistics for the S138A Pin1 PPIase mutant are summarized in Table S1. The programs PyMOL (DeLano Scientific, San Carlos, CA, USA) and MOLMOL [51] were used for structure visualization and figure preparation. The resonance assignments and structural data for the S138A Pin1 PPIase mutant have been deposited in the BioMagResBank (BMRB) and Protein Data Bank (PDB) databases, respectively. The BMRB accession code is 36014. The PDB ID is 5GPH.

### 4.4. Measuring the Cis−Trans Isomerization Rate for a Phosphor-Peptide

The *cis*−*trans* isomerization rates of the S138A Pin1 PPIase domain were measured by 2D ^1^H,^1^H-EXSY spectroscopy [23,33]. The sample contained 2 mM phosphor-peptide Cdc25C (EQPLpTPVTDL, where pT represents a phosphorylated threonine) with 50 μM of the S138A PPIase domain. The sample solution contained 50 mM Tris-HCl (pH 6.8), 1 mM DTT and 0.03% NaN_3_. The experiments were carried out at 295 K. The mixing times (*t*_mix_) were 2, 5, 10, 15, 25, 35, 50 (twice), 75, 100 (twice), 200, 300 and 400 ms. The net exchange rate, *k*_EX_, was determined using the equation below (Equation (4)) to fit the ratios of the *trans*-to-*cis* exchange cross-peaks against the *trans* diagonal peaks:

(4) $$\mathrm{ratio}\left(t_{\mathrm{mix}}\right)=\frac{\left[1-e^{-\left(k_{\mathrm{CT}}+k_{\mathrm{TC}}\right)t_{\mathrm{mix}}}\right]k_{\mathrm{TC}}}{k_{\mathrm{CT}}+k_{\mathrm{TC}}e^{-\left(k_{\mathrm{CT}}+k_{\mathrm{TC}}\right)t_{\mathrm{mix}}}}$$

where *k*_CT_ and *k*_TC_ are adjustable parameters in fitting and the net exchange rate, *k*_EX_, is defined as *k*_EX_ = *k*_CT_ + *k*_TC_, where *k*_CT_ and *k*_TC_ are the exchange rates from *cis* to *trans* and *trans* to *cis*, respectively [23]. Uncertainties in the rate constants were estimated by Monte Carlo simulations using the duplicated data. In estimating the *k*_EX_ values, we simultaneously used the buildup profiles from the signals for pT5 CH_3_, pT5 HN, and V7 HN for fitting, as in a global fitting manner. The phosphor-peptide Cdc25C was purchased from Funakoshi (Tokyo, Japan) and used without further purification.

### 4.5. NMR Spin Relaxation Experiments

All backbone ^15^N *R*_1_ and *R*_2_ relaxation rates and steady state heteronuclear ^15^N NOE (hNOE) data were collected on the same 700.33 MHz NMR spectrometer at 299 K [42]. Each peak intensity was measured by averaging over the signal intensities at the peak center and its eight surrounding points (nine-point averaging) using an in-house program; each peak center was found by the SPARKY “pc” function (T.D. Goddard and D. G. Kneller, SPARKY 3, University of California, San Francisco, CA, USA). The delays for *R*_1_ measurements (*t*_relax_) were 10.3 (twice), 153.9, 307.9, 461.8, 615.7 (twice), 769.6, 923.6, 1128.8 and 1539.3 ms, whereas the delays for *R*_2_ (*t*_relax_) were 0.0, 16.0 (twice), 40.0, 80.0 (twice) and 160.0 ms. Spectra for *R*_1_ and *R*_2_ were collected in an interleaved manner. For measuring hNOEs, we recorded an interleaved pair of spectra in which ^1^H saturation of 3 s was applied alternatively with the relaxation delay set to 2 s. *R*_1_ and *R*_2_ relaxation rate constants for each signal were determined using the modelXY TCL built-in function of NMRPipe [43]. Uncertainties for *R*_1_ and *R*_2_ were estimated in a Monte Carlo manner using the duplicated data points. The uncertainty for each hNOE value was evaluated using the standard deviation of the noise on a spectral region with no peaks, which was obtained by the NMRPipe built-in module [43]. The reduced spectral density functions, including *J*_eff_(0), *J*(*ω_N_*) and *J*(*ω_h_*), were calculated using the software suite RELAX [52,53]; *ω_h_* represents the scaled ^1^H angular velocity 0.87*ω_H_* with *ω_H_* being 2π × 700.33 × 10^6^ rad s^−1^ in the present work.

### 4.6. H/D Exchange Rates

The amide proton/deuteron exchange (H/D exchange) rates were measured with extensively lyophilized samples. The sample solution (S138A Pin1 PPIase domain in 50 mM sodium phosphate (pH 6.6), 100 mM Na_2_SO_4_, 5 mM EDTA, 1 mM DTT and 0.03% NaN_3_) was rapidly frozen in liquid nitrogen and placed in a vacuum chamber for approximately 12 h. Just prior to recording a series of 2D ^1^H–^15^N HSQC spectra the lyophilized sample was dissolved in the same volume of D_2_O as it was before lyophilization. NMR measurements were initiated within 10 min after dissolving the sample. One spectrum was collected for 35 min and 35 data sets were collected sequentially. No apparent spectral difference was observed between the data before and after lyophilization, which ensures the preparation had no impact on the structure of the mutant. H/D exchange rates were determined through the peak intensities of a series of 2D spectra, as described in our previous work [13].

### 4.7. Isothermal Titration Calorimetry Experiments

Isothermal titration calorimetry (ITC) experiments consisted of a series of 1.5 μL injections of 10 mM Cdc25C phosphor-peptide into 200 µL 0.2 mM wild-type or S138A Pin1 PPIase domain solution in the thermostatic cell with an initial delay of 60 s, a 3 s duration of injection and a spacing between injections of 150 s using a Microcal Auto iTC200 instrument (Malvern, Worcestershire, UK). The protein concentrations were determined by absorption at 280 nm using a molar extinction coefficient of 6.99 mM^−1^ cm^−1^. All concentrations were measured on a Nanodrop 2000 (Thermo Fisher Scientific, Waltham, MA, USA). The phosphopeptide compound was weighed using an electronic microbalance CP225D (Sartorius, Göttingen, Germany). The collected data were analyzed with the Microcal ORIGIN software (Malvern). The corrected binding isotherms were fitted using a single-site with the stoichiometry fixed to *n* = 1, because the system in this study indicated low binding-affinity [31,32]. All experiments were repeated in triplicate.

### 4.8. Deuterium Isotope Shift for Histidine Imidazole ^15^N Chemical Shifts

The imidazole ^15^N chemical shifts were measured using multiple-bond 2D ^1^H–^15^N HSQC spectra for sample solutions containing 6%, 50% and 100% D_2_O [38]. The sample was prepared from the lyophilized protein solution containing 50 mM sodium phosphate buffer (pH 6.6) and 100 mM sodium sulfate. To exclusively eliminate the proton, the lyophilized sample was dissolved in D_2_O and then subjected to further lyophilization. The procedure was repeated twice to extensively purge protons from the sample. In the final sample solution containing different amounts of D_2_O, 5 mM EDTA, 1 mM DTT and 0.03% NaN3 were added. All NMR data were collected at 299 K.

## 5. Conclusions

In this report, the functional role of dynamic allostery in the PPIase domain of Pin1 was examined. The S138A mutation rearranged residue-residue contacts near the mutation site, which changed the conformational dynamics of the catalytic β1-α2 and α2-α3 loops that are both distal from the mutation site (Figure 9A). Despite the significant change in the dynamics of the catalytic loops, their structures remained unchanged, indicative of dynamic allostery at play [19]. The S138A mutation facilitated dynamic allostery in the PPIase domain in a similar way as envisaged by the interdomain contact in the full-length Pin1 [17]. Thus, the S138A mutation mimics the interdomain contact by replacing the link between the catalytic site and the interdomain contact interface where S138 functions in a dynamic allosteric manner, as found in the full-length Pin1 [17].

The S138A mutation caused the α2-α3 loop to be more rigid than the wild-type domain to stabilize the hydrogen bond between C113 and H59 in the hydrogen bond network (Figure 9B). Protonation of the thiolate of C113 initiates substrate isomerization bound to the catalytic site in the PPIase domain [29]. Stabilization of the hydrogen bond formed between the thiolate S^γ^ in C113 and the N^ε2^ in H59 (Figure 9B), therefore, retards the isomerization process; the S138A mutation reduced the isomerization rate by ~20% when compared with that of the wild-type PPIase domain (Table 1).

In summary, using the S138A mutant, we revealed that dynamic allostery affects the catalytic reaction through rearrangement of the hydrogen bond network within the catalytic site (Figure 9). Previous research has shown that the interdomain contact reduces the conformation dynamics of the PPIase domain by dynamic allostery between the interdomain interface and the catalytic site [17,26,41]. Based on previous observations, researchers have proposed that the reduced conformation dynamics in the catalytic site compensates for the entropy cost upon substrate binding to the PPIase domain and thus improves isomerization activity [17,26,41]. Our results provide further data that describe how the dynamic allostery mechanism affects the reaction steps to isomerize the Pro peptide bond in the substrate. Although the S138A mutant caused functionally negative dynamic allostery, the mechanism presented in this work will advance our understanding of how the interdomain contact uses dynamic allostery to enhance the catalytic activity of Pin1.

In this sense, it is intriguing to note that S138E mutation destabilized the PPIase domain structure. This observation may suggest that positive dynamic allostery may destabilize the hydrogen bonding network, especially at the bond formed at C113–H59, to facilitate the rearrangement in the hydrogen bonds in the network (Figure 9B).

## Figures and Tables

**Table 1:** Global structural stabilities and the *cis*-*trans* isomerization rates for a series of Pin1 peptidyl-prolyl isomerase (PPIase) mutants.

Pin1 PPIase	Denaturation Temperature (°C) ^a^	Exchange Rate from *cis* to *trans* (*k*_CT_) (s^−1^) ^b^	Exchange Rate from *trans* to *cis* (*k*_TC_) (s^−1^) ^b^
Wild-type ^c^	49.4	51.6 ± 1.9	6.6 ± 2.1
S138A	55.8	43.8 ± 0.7	3.7 ± 2.0
C113D ^c^	46.2	0.7 ± 0.5	0.1 ± 0.0
C113A ^c^	43.2	1.0 ± 0.2	0.1 ± 0.1
C113S ^c^	43.6	Not detected	Not detected

^a^ Denaturation temperature was estimated by CD molar ellipticity at 222 nm; ^b^ Isomerization exchange rates were directly determined using Exchange Spectroscopy (EXSY) experiments; ^c^ These values were determined in our previous studies [13,30].

## Supplementary Materials

The following materials are available online, Figure S1. Structural and backbone chemical shift changes induced by the S138A mutation; Figure S2. Comparison of phosphorylated peptide binding ability between the wild-type protein and the S138A mutant; Figure S3. Comparison of hetero-nuclear NOEs between the S138A mutant and the wild-type Pin1 PPIase domain; Figure S4. Graphical representation of the correlation between *J*(0) and *J*(*ω_N_*); Figure S5. A 2D ^1^H−^15^N multi-bond HSQC spectrum for the imidazole rings of the S138A Pin1 PPIase domain mutant; Table S1: Structural statistics of the final 10 structures of the S138A Pin1 PPIase mutant; Table S2: The relaxation parameters for the W73 side chain (Nε); Table S3. Observed NOEs related to W73 (See Figure S1A inset); Supplementary References.
