# Supplementary material for: Dynamic Allostery Modulates Catalytic Activity by Modifying the Hydrogen Bonding Network in the Catalytic Site of Human Pin1
Source: Molecules. 2017 Jun 15;22(6):992. doi: 10.3390/molecules22060992 (PMC6152768; doi:10.3390/molecules22060992)
Supplement: Supplementary file 1 [file molecules-22-00992-s001.pdf]

Supplementary Material

Dynamic Allostery Modulates Catalytic Activity by Modifying the Hydrogen-Bonding Network in the Catalytic Site of Human Pin1

Jing Wang[1,†], Ryosuke Kawasaki[1,†], Jun-ichi Uewaki[2], Arif U.R. Rashid[1], Naoya Tochio[2] and Shin-ichi Tate[1,2,*]

[1]Department of Mathematical and Life Sciences, School of Science, Hiroshima University, 1-3-1 Kagamiyama, Higashi-Hiroshima 739-8526, Japan
[2]Research Center for the Mathematics on Chromatin Live Dynamics (RcMcD), Hiroshima University, 1-3-1 Kagamiyama, Higashi-Hiroshima 739-8526, Japan

[†]These authors contributed equally to this work.

*Correspondence:

Shin-ichi Tate

Department of Mathematical and Life Sciences,

School of Science, Hiroshima University

1-3-1 Kagamiyama, Higashi-Hiroshima 739-8526, Japan.

Tel: +81-82-424-7387

e-mail: tate@hiroshima-u.ac.jp

Figure Captions

Figure S1. Structural and backbone chemical shift changes induced by the S138A mutation. (A) Structural superposition of the wild-type (grey) [1] and S138A mutant (red). The basic triad and W73 are shown as stick models. The S138A mutation site is indicated as a sphere in red. In the inset, the NOEs related to W73 in the S138A mutant are shown as dashed yellow lines: the NOE connectivities from W73 are listed in Table S3. (B) Normalized $^{1}H_{N}$ and ^{15}N backbone chemical shift differences between the wild-type [2] and the S138A mutant residues. The normalized chemical shift difference for each residue is defined as $\Delta\delta = [(\Delta\delta^{1}H)^{2} + (\Delta\delta^{15}N/5)^{2}]^{1/2}$, where $\Delta\delta^{1}H$ and $\Delta\delta^{15}N$ are the chemical shift differences in $^{1}H_{N}$ and ^{15}N dimensions, respectively [3]. (C) The NOE signals observed between C113 H^{N} and W73 $H^{\varepsilon 1}$. Signals with asterisks come from the other residues at different ^{15}N planes in a 3D ^{15}N-editted NOESY spectrum.

Figure S2. Comparison of phosphorylated peptide binding ability between the wild-type protein and the S138A mutant. Isothermal calorimetric titration data at 298 K for the wild-type Pin1-PPIase (left) and the S138A mutant (right) are shown. The red solid lines drawn in the ΔH plot (bottom panels) represent the best-fit model function assuming a 1:1 stoichiometry.

Figure S3. Comparison of heteronuclear NOEs between the S138A mutant and the wild-type Pin1 PPIase domain. (A) The heteronuclear NOEs for the S138A mutant (red) and those for the wild-type Pin1 PPIase domain (black) [2]. (B) Difference between the ^{15}N-^{1}H heteronuclear NOEs (hNOEs) for the S138A mutant and those for the wild-type Pin1 PPIase domain [2]. The difference, ΔhNOE, is defined as $\Delta hNOE = hNOE^{S138A} - hNOE^{wild\text{-}type}$. The region F103–A116 exhibited reduced $\Delta J(\omega_{h})$ values relative to other residues are orange-boxed.

Figure S4. Graphical representation of the correlation between $J(0)$ and $J(\omega_N)$ [4]. The simple correlation between $J(0)$ and $J(\omega_N)$ described in the main text eq. (1) is shown as the blue curve. Point *a* represents a residue with the N–H bond motion completely dominated by τ_c; *b* represents a residue with significant rapid internal motion due to the local correlation time, τ_e; *c* represents a residue with the N–H bond motion mainly dominated by rapid internal motion; and *d* represents a residue with slow conformational fluctuations.

Figure S5. A 2D $^1H-^{15}N$ multi-bond HSQC spectrum [5] for the imidazole rings of the S138A Pin1 PPIase domain mutant. The set of signals marked with an asterisk represent the histidine located within the N-terminal GSHM segment, which is present because of the expression construct.

Table S1. Structural statistics of the final 10 structures of S138A Pin1 PPIase mutant.

Completeness of resonance assignments (%)[a]	
Backbone	97.8
Side chain	99.3
Aromatic	100
Stereospecific methyl	100
Conformationally restricting restraints	
Distance restraints	
Total	2,691
Intraresidue ($i = j$)	543
Sequential ($\|i - j\| = 1$)	609
Medium range ($1 < \|i - j\| < 5$)	528
Long range ($\|i - j\| \geq 5$)	1,011
Dihedral angle restraints[b]	102
Hydrogen-bond restraints	0
Disulfide restraints	0
No. of restraints per residue	23.0
No. of long-range restraints per residue	8.6
Residual restraint violations	
Average no. of distance violations per structure	
0.1−0.3 Å	2.4
0.3−0.5 Å	0.1
> 0.5 Å	0
Average no. of dihedral angle violations per structure	
> 5°	0
Model quality[c]	
RMSD backbone atoms (Å)[d]	0.7
RMSD heavy atoms (Å)[d]	1.1
RMSD bond lengths (Å)	0.007
RMSD bond angles (°)	0.6
MolProbity Ramachandran statistics[c,d]	
Most favored regions (%)	94.7
Allowed regions (%)	5.3
Disallowed regions (%)	0
Global quality scores (raw / Z score)[c]	
Verify3D	0.40 / -0.96
Prosall	0.72 / 0.29
PROCHECK (ϕ-ψ)[d]	-0.28 / -0.79
PROCHECK (all)[d]	-0.19 / -1.12
MolProbity clash score	16.68 / -1.34
Model contents	
Total no. of residues	117
BMRB accession number	36014
PDB ID code	5GPH

[a]The number excluding highly exchangeable protons, nitrogens bound with highly exchangeable protons, nonprotonated carbons and nitrogens.
[b]The angle restraints were derived from TALOS+ [6] with the angle ranges ± 30°.
[c]Calculated using PSVS version 1.5 [7].
[d]For residues P52-S111, S114-S126, Q129-M146, and P149-T162 as the ordered residues estimated by PSVS.

Table S2. The relaxation parameters for the side chain of W73 (Nε).

	$J(0)$ [ns]	$J(\omega_N)$ [ns]	$J(\omega_h)$ [ps]	R_1 [s^{-1}]	R_2 [s^{-1}]	*hNOE* [ratio]
Wild-type[a]	7.72 ± 0.03	0.190 ± 0.002	3.34 ± 0.18	0.92 ± 0.01	24.52 ± 0.08	0.77 ± 0.01
S138A	6.31 ± 0.03	0.232 ± 0.002	3.97 ± 0.26	1.12 ± 0.01	20.23 ± 0.09	0.77 ± 0.02
C113D[a]	6.62 ± 0.05	0.218 ± 0.003	3.51 ± 0.20	1.05 ± 0.01	21.15 ± 0.16	0.79 ± 0.01
C113A[a]	9.95 ± 0.18	0.204 ± 0.007	3.51 ± 0.32	0.98 ± 0.03	31.49 ± 0.58	0.77 ± 0.02
C113S[a]	6.67 ± 0.06	0.196 ± 0.006	2.97 ± 0.27	0.94 ± 0.03	21.24 ± 0.19	0.80 ± 0.02

Side chain relaxation for W73 was analyzed in a manner identical to that for the backbone amide group except using −107.7 ppm as the ^{15}N CSA [8].
[a]These values were determined using data in our previous studies [1,9].

Table S3. Observed NOEs related to W73 (See Figure S1A inset).

Residue1	Atom1	Residue2	Atom2
W73	H^{N}	S72	H^{N}
W73	H^{N}	S72	$H^{\beta\#}$
W73	$H^{\varepsilon1}$	S72	$H^{\beta\#}$
W73	H^{N}	R74	H^{N}
W73	$H^{\delta1}$	R74	H^{N}
W73	$H^{\delta1}$	R74	$H^{\gamma\#}$
W73	$H^{\varepsilon1}$	R74	$H^{\gamma\#}$
W73	$H^{\varepsilon3}$	R74	H^{α}
W73	$H^{\zeta2}$	R74	$H^{\delta\#}$
W73	$H^{\zeta2}$	R74	$H^{\delta\#}$
W73	$H^{\zeta2}$	R74	$H^{\gamma\#}$
W73	$H^{\zeta3}$	R74	H^{α}
W73	$H^{\eta2}$	Q109	H^{α}
W73	$H^{\eta2}$	Q109	$H^{\gamma\#}$
W73	$H^{\zeta2}$	Q109	H^{α}
W73	$H^{\varepsilon1}$	D112	H^{α}
W73	$H^{\varepsilon1}$	C113	H^{N}
W73	$H^{\delta1}$	S114	$H^{\beta\#}$
W73	$H^{\delta1}$	A116	$H^{\beta\#}$
W73	$H^{\varepsilon1}$	A116	$H^{\beta\#}$
W73	$H^{\eta2}$	A116	$H^{\beta\#}$
W73	$H^{\zeta2}$	A116	$H^{\beta\#}$

# represents ambiguous methylene protons.

Supplementary References

1. Xu, N.; Tochio, N.; Wang, J.; Tamari, Y.; Uewaki, J.-i.; Utsunomiya-Tate, N.; Igarashi, K.; Shiraki, T.; Kobayashi, N.; Tate, S.-i. The c113d mutation in human pin1 causes allosteric structural changes in the phosphate binding pocket of the ppiase domain through the tug of war in the dual-histidine motif. *Biochemistry* **2014**, *53*, 5568-5578.
2. Xu, N.; Tochio, N.; Wang, J.; Tamari, Y.; Uewaki, J.; Utsunomiya-Tate, N.; Igarashi, K.; Shiraki, T.; Kobayashi, N.; Tate, S. The c113d mutation in human pin1 causes allosteric structural changes in the phosphate binding pocket of the ppiase domain through the tug of war in the dual-histidine motif. *Biochemistry* **2014**, *53*, 5568-5578.
3. Hall, D.A.; Vander Kooi, C.W.; Stasik, C.N.; Stevens, S.Y.; Zuiderweg, E.R.; Matthews, R.G. Mapping the interactions between flavodoxin and its physiological partners flavodoxin reductase and cobalamin-dependent methionine synthase. *Proc Natl Acad Sci U S A* **2001**, *98*, 9521-9526.
4. Krizova, H.; Zidek, L.; Stone, M.J.; Novotny, M.V.; Sklenar, V. Temperature-dependent spectral density analysis applied to monitoring backbone dynamics of major urinary protein-i complexed with the pheromone 2- sec-butyl-4,5-dihydrothiazole. *Journal of biomolecular NMR* **2004**, *28*, 369-384.
5. Pelton, J.G.; Torchia, D.A.; Meadow, N.D.; Roseman, S. Tautomeric states of the active-site histidines of phosphorylated and unphosphorylated iiiglc, a signal-transducing protein from escherichia coli, using two-dimensional heteronuclear nmr techniques. *Protein science : a publication of the Protein Society* **1993**, *2*, 543-558.
6. Shen, Y.; Delaglio, F.; Cornilescu, G.; Bax, A. Talos+: A hybrid method for predicting protein backbone torsion angles from nmr chemical shifts. *J Biomol NMR* **2009**, *44*, 213-223.
7. Bhattacharya, A.; Tejero, R.; Montelione, G.T. Evaluating protein structures determined by structural genomics consortia. *Proteins: Structure, Function, and Bioinformatics* **2007**, *66*, 778-795.
8. Miloushev, V.Z.; Bahna, F.; Ciatto, C.; Ahlsen, G.; Honig, B.; Shapiro, L.; Palmer, A.G., 3rd. Dynamic properties of a type ii cadherin adhesive domain: Implications for the mechanism of strand-swapping of classical cadherins. *Structure (London, England : 1993)* **2008**, *16*, 1195-1205.
9. Wang, J.; Tochio, N.; Kawasaki, R.; Tamari, Y.; Xu, N.; Uewaki, J.-i.; Utsunomiya-Tate, N.; Tate, S.-i. Allosteric breakage of the hydrogen bond within the dual-histidine motif in the active site of human pin1 ppiase. *Biochemistry* **2015**, *54*, 5242-5253.

A

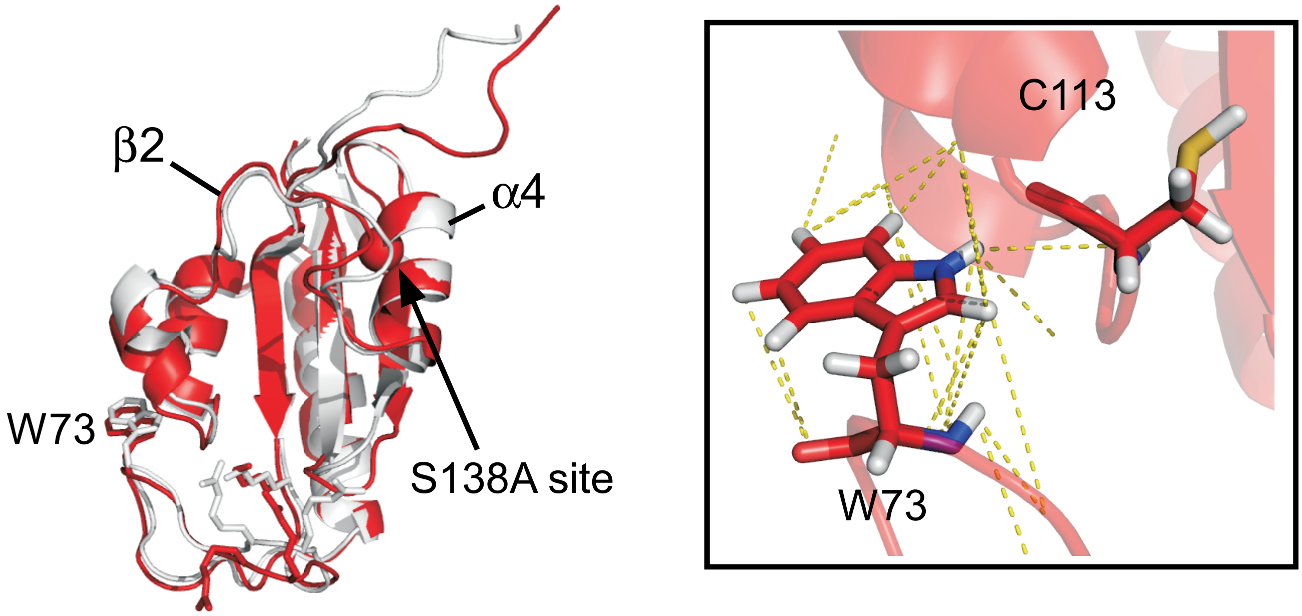
β2
α4
W73
S138A site
C113
W73

B

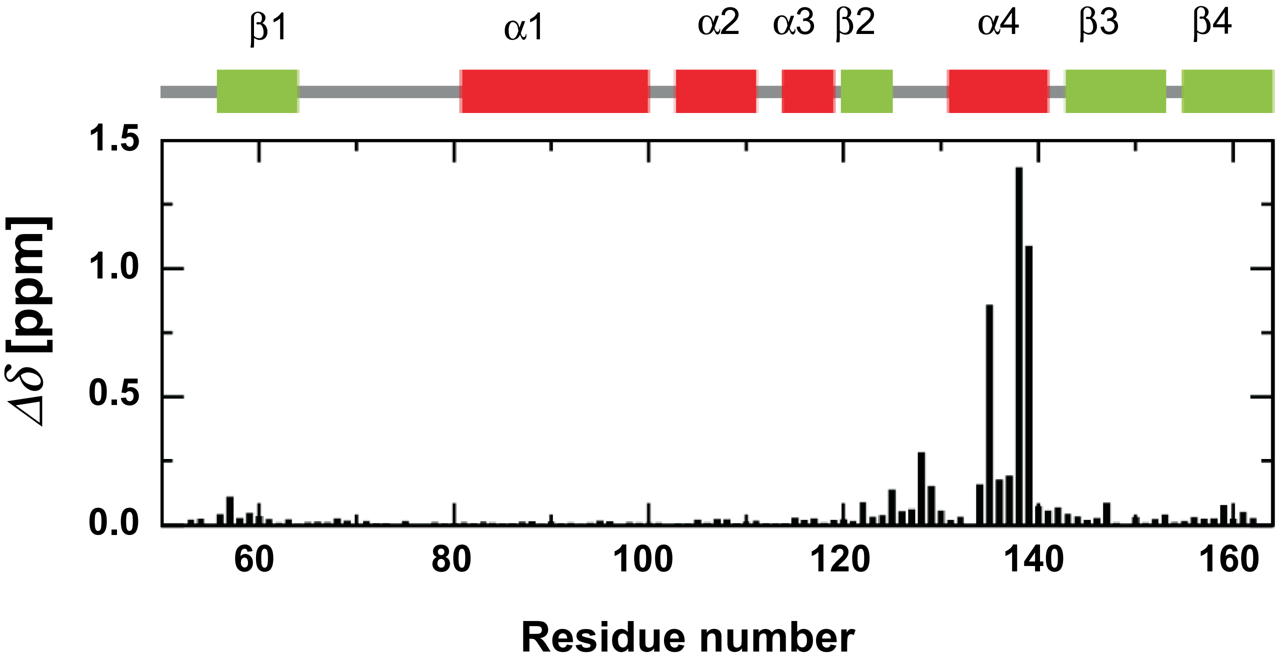
β1
α1
α2
α3
β2
α4
β3
β4
1.5
1.0
0.5
0.0
Δδ [ppm]
60
80
100
120
140
160
Residue number

Figure S1

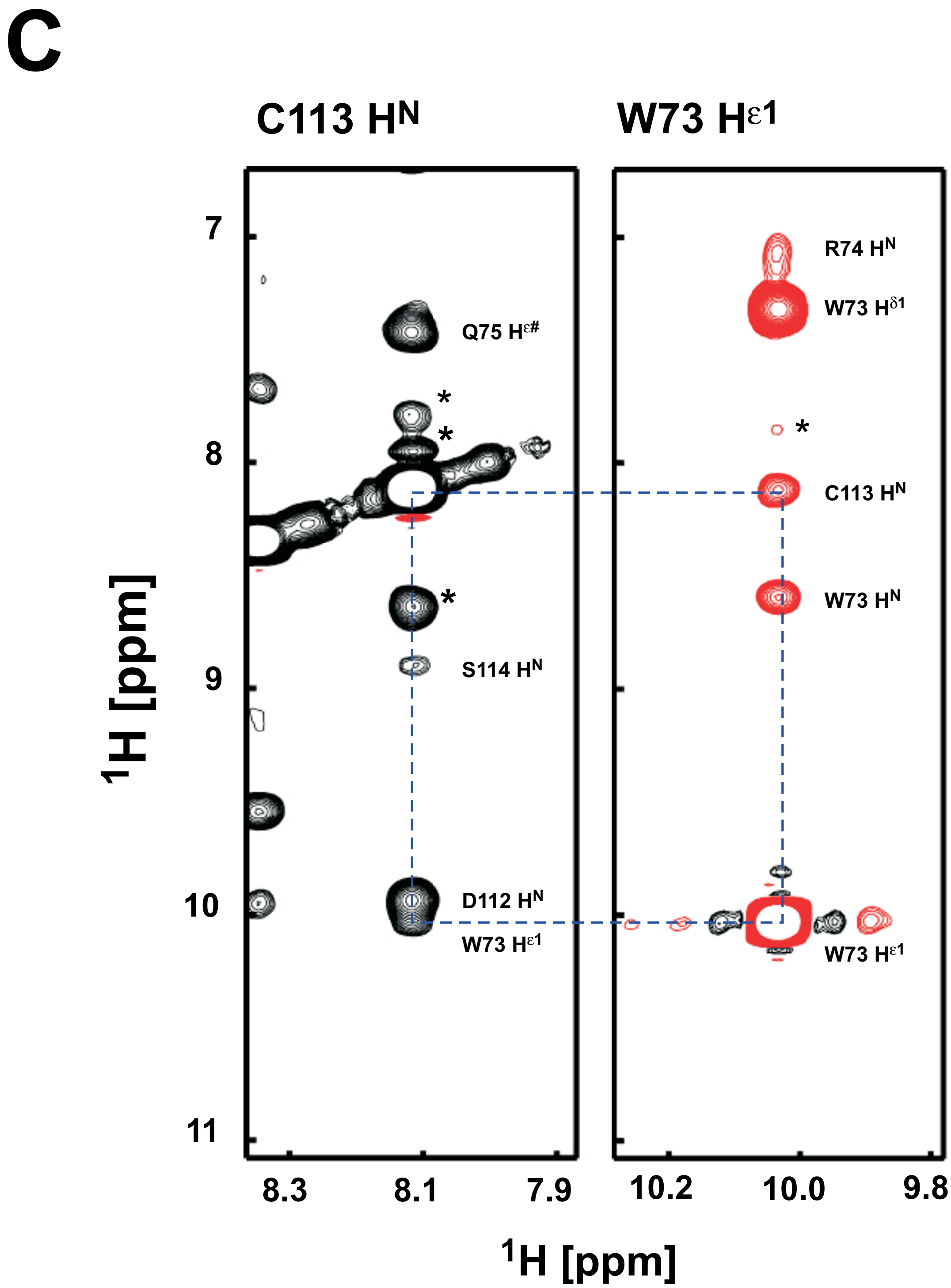
C
C113 H^N
W73 Hε1
Q75 Hε#
*
*
*
S114 H^N
D112 H^N
W73 Hε1
R74 H^N
W73 Hδ1
*
C113 H^N
W73 H^N
W73 Hε1
7
8
9
10
11
8.3
8.1
7.9
10.2
10.0
9.8
1H [ppm]
1H [ppm]

Figure S1

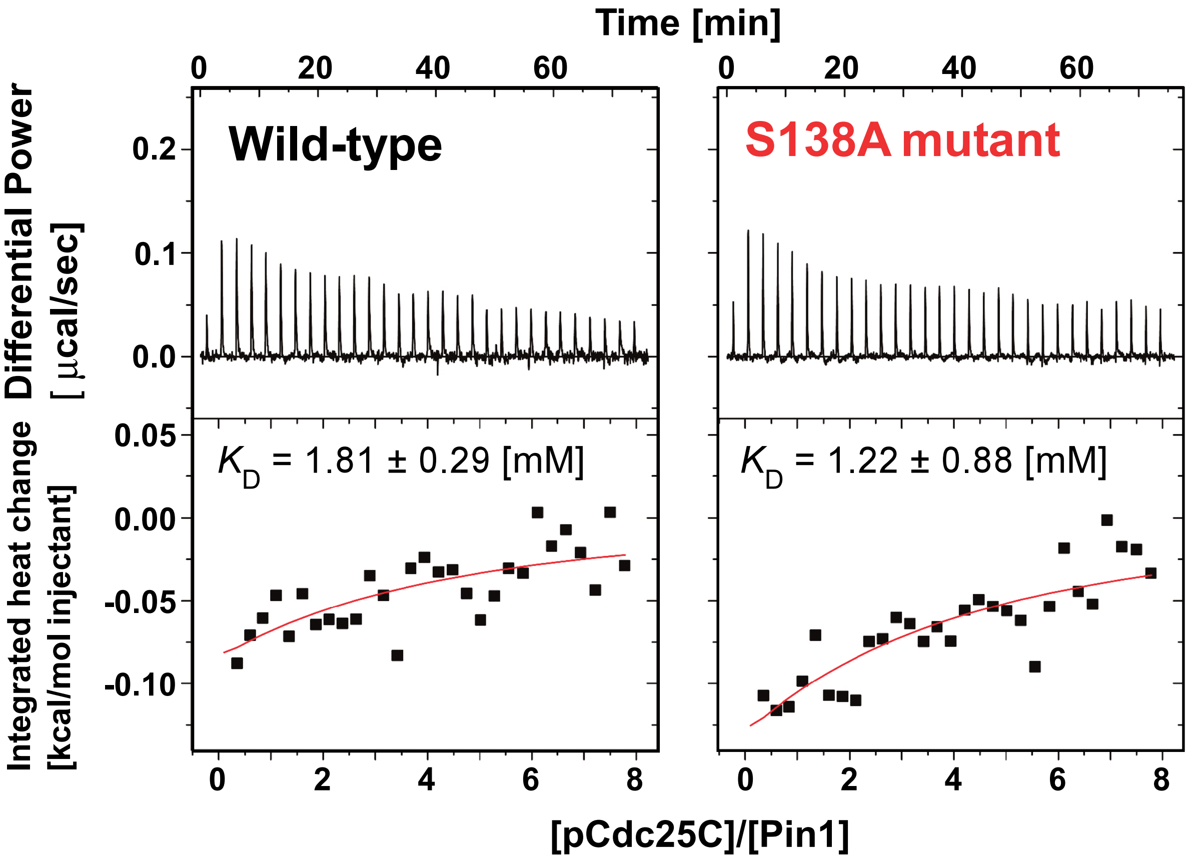

Time [min]
0
20
40
60
0
20
40
60
Wild-type
S138A mutant
Differential Power
[μcal/sec]
0.2
0.1
0.0
Integrated heat change
[kcal/mol injectant]
0.05
0.00
-0.05
-0.10
K_D = 1.81 ± 0.29 [mM]
K_D = 1.22 ± 0.88 [mM]
0
2
4
6
8
0
2
4
6
8
[pCdc25C]/[Pin1]

Figure S2

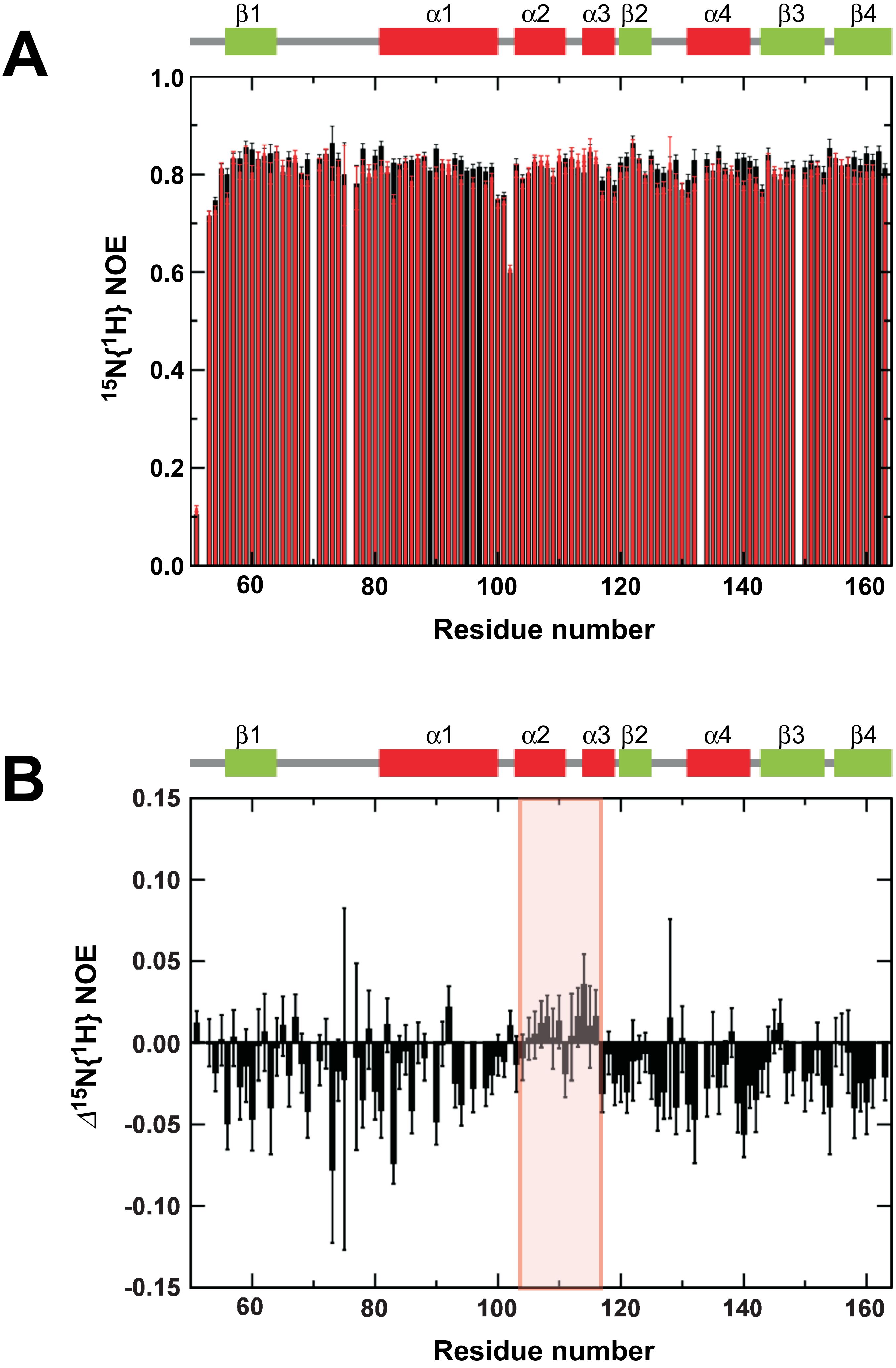

A
β1
α1
α2
α3
β2
α4
β3
β4
1.0
0.8
0.6
0.4
0.2
0.0
^{15}N{^{1}H} NOE
60
80
100
120
140
160
Residue number
B
0.15
0.10
0.05
0.00
-0.05
-0.10
-0.15
Δ^{15}N{^{1}H} NOE

Figure S3

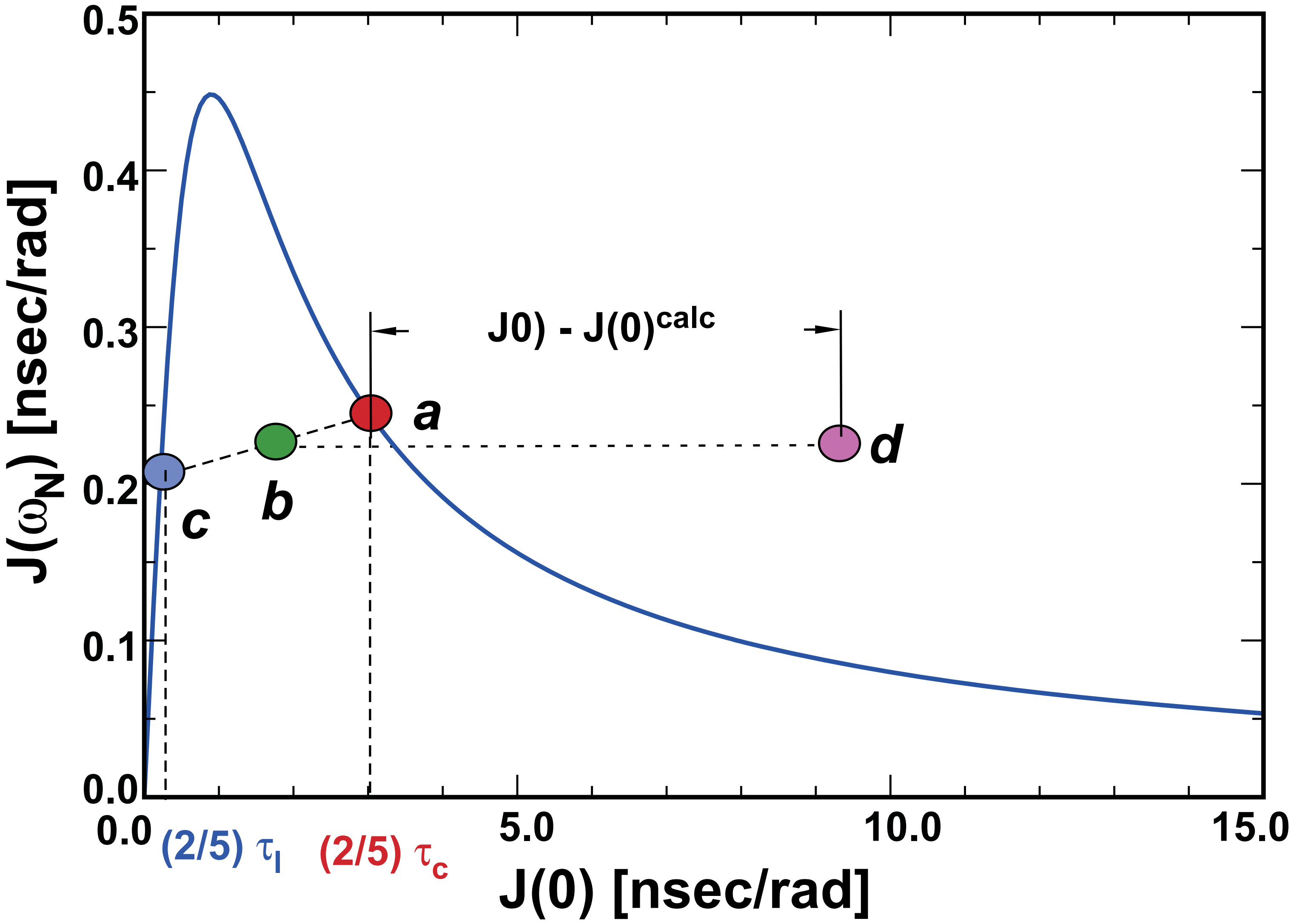

0.5
0.4
0.3
0.2
0.1
0.0
J(ω_N) [nsec/rad]
J0) - J(0)^calc
a
b
c
d
0.0
(2/5) τ_I
(2/5) τ_c
5.0
10.0
15.0
J(0) [nsec/rad]

Figure S4

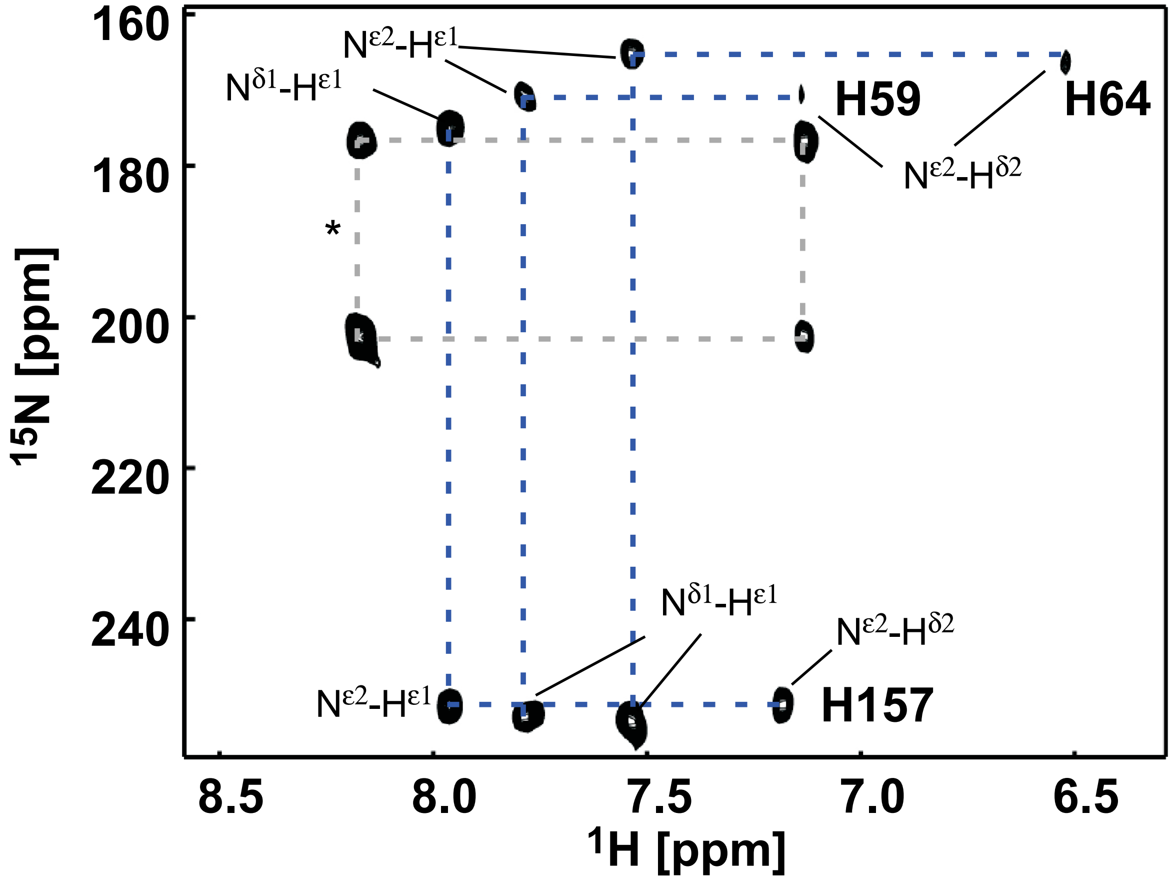

160
180
200
220
240
^{15}N [ppm]
8.5
8.0
7.5
7.0
6.5
^{1}H [ppm]
$N^{\varepsilon2}$-$H^{\varepsilon1}$
$N^{\delta1}$-$H^{\varepsilon1}$
H59
H64
$N^{\varepsilon2}$-$H^{\delta2}$
*
$N^{\delta1}$-$H^{\varepsilon1}$
$N^{\varepsilon2}$-$H^{\delta2}$
$N^{\varepsilon2}$-$H^{\varepsilon1}$
H157

Figure S5
